# Proteolytic Activity and Volatile Compound Formation by *Staphylococcus warneri*, *Rothia uropygioeca*, and *Enterococcus lactis* in Pea Protein Fermentations

**DOI:** 10.3390/foods15213870

**Published:** 2026-10-29

**Authors:** José María Martín-Miguélez, Cristina Castaño, Irene Martín, Josué Delgado, Juan J. Córdoba

**Affiliations:** Higiene y Seguridad Alimentaria, Instituto Universitario de Investigación de Carne y Productos Cárnicos (IProCar), Facultad de Veterinaria, Universidad de Extremadura, Avda. de las Ciencias s/n, 10003 Cáceres, Spain; jmmm@unex.es (J.M.M.-M.); cristinacs@unex.es (C.C.); iremartint@unex.es (I.M.); jcordoba@unex.es (J.J.C.)

**Keywords:** plant-based analog, free amino acids, biogenic amines, proteolytic agar, lypolytic agar, pea protein isolate, textured pea protein

## Abstract

The transition toward clean label, plant-based foods has intensified the search for natural fermentation strategies to reduce off-flavors, while improving taste and nutritional quality of plant proteins. This study evaluated 142 microorganisms isolated from animal-derived ripened foods, selecting four proteolytic Gram-positive cocci (*Staphylococcus warneri* GC 13 and 28, *Rothia uropygioeca* GC 15, and *Enterococcus lactis* GC 40) for fermenting pea protein isolate and textured pea protein as plant-based model substrates. Fermentations at 25 °C for 48 h significantly modified microbiological and physicochemical parameters in a matrix-dependent manner, with textured pea protein supporting higher lactic acid bacteria growth and stronger acidification than pea protein isolate. Free amino acid profiling demonstrated strain- and matrix-dependent proteolytic activity, with GC 13 and 15 promoting the highest total free amino acids in pea protein isolate, while GC 15, 28, and 40 markedly increased multiple amino acids in textured pea protein. Biogenic amine analysis revealed that tyramine was the predominant amine, reaching 162–212 ng/g in inoculated textured pea protein and 348 ng/g in uninoculated controls, whereas only trace levels were detected in the pea protein isolate. Volatile organic compound analysis grouped compounds into three clusters, evidencing a reduction in native beany aldehydes and the formation of alcohols, ketones, and acids, particularly in textured protein, indicating substantial reshaping of the aroma profile. Overall, the results showed that the evaluated strains can be exploited as clean-label tools to enhance proteolysis, attenuate off-flavors, and maintain acceptable biogenic amine levels in pea protein matrices, with *R. uropygioeca* 15 emerging as a promising candidate for plant protein fermentation.

## 1. Introduction

The global food industry is undergoing a transformative shift driven by rising consumer demand for clean-label products that prioritize transparency, minimal processing, and the absence of additives [1]. This consumer-driven trend has prompted food manufacturers to reformulate traditional products by replacing synthetic ingredients with natural alternatives that meet health, sustainability, and ethical expectations [2,3]. In this context, microbial fermentation emerges as a sophisticated biotechnological approach that aligns with clean label principles while addressing critical challenges in plant-based food production [4,5].

Plant-based proteins have gained attention as sustainable alternatives to animal-derived proteins [6]. However, their widespread adoption faces significant sensory barriers, particularly the presence of undesirable off-flavors, such as beany and bitter flavors, which negatively impact consumer acceptance [7,8,9]. These characteristic flavors originate primarily from lipid oxidation products, including aldehydes and ketones, which interact with proteins and phytochemicals in the plant matrix. Traditional approaches to flavor improvement often rely on masking agents or synthetic flavor enhancers, which contradict the basic principles of clean-label production. However, fermentation has been studied as a valuable strategy to remove off-flavors from plant-based products, avoiding use of synthetic flavor enhancers [10].

Proteolytic microorganisms, such as bacteria and yeasts, present a natural solution to enhance flavor profiles in plant-based matrices without requiring artificial additives [11,12,13]. The proteolytic activity of these microorganisms extends beyond simple protein degradation, generating compounds that contribute to umami, kokumi, and savory taste sensations while simultaneously reducing bitter and off-flavor notes [14]. The fermentation process offers additional advantages beyond flavor development, including enhanced nutritional bioavailability, improved protein digestibility, and the production of bioactive peptides with antioxidant, antihypertensive, and antimicrobial properties [15,16,17,18]. From a sustainability perspective, microbial fermentation represents an environmentally responsible biotechnology that minimizes waste, reduces reliance on chemical additives, and transforms agricultural by-products into valuable functional ingredients [5,19].

Despite the demonstrated potential of proteolytic microorganisms for plant protein valorization, systematic understanding of strain-specific proteolytic profiles, optimization of fermentation parameters, and elucidation of flavor compound formation mechanisms remain areas requiring further investigation. Furthermore, much of the proteolytic activity has been demonstrated in sterile plant matrices, but not in real, unsterilized plant matrices, which may contain contaminating microbiota.

The present study explores the use of selected proteolytic microorganisms in unsterilized plant-based matrices as a clean-label strategy for natural flavor development, eliminating the need for synthetic additives while enhancing nutritional value and sensory quality. Through microbial fermentation, we aim to demonstrate a sustainable biotechnological approach that meets contemporary consumer demands for transparency, health, and environmental responsibility in food production.

## 2. Materials and Methods

### 2.1. Microbial Cultures

A total of 142 microorganisms from the collection of the Food Hygiene and Safety Research Group at the University of Extremadura were utilized in the present study. Specifically, one hundred bacteria and forty-two yeasts isolated from animal-derived ripened products were used. The microbial cultures were revived from frozen stocks (−80 °C) maintained in their respective growth media supplemented with 20% (*w/v*) glycerol (Thermo Fisher Scientific, Waltham, MA, USA). For inoculum preparation, Gram-positive cocci (GC), yeasts, and lactic acid bacteria (LAB) strains were cultivated in Brain Heart Infusion (BHI; Conda Pronadisa, Madrid, Spain), Malt Extract Broth (MEB; 20 g of malt extract (Conda Pronadisa), 20 g of glucose (Labbox Labware S.L., Barcelona, Spain), 1 g of bacteriological peptone (Conda Pronadisa) in 1 L of distilled water), and Man, Rogosa, and Sharpe broth (MRS; Conda Pronadisa), respectively, at 30 °C for 48 h.

Following incubation, cell suspensions were centrifuged in 50 mL polypropylene tubes (Scharlau Chemie S.A., Barcelona, Spain) at 10,621× *g* for 5 min. The supernatant was discarded, and the resulting pellet was resuspended in Phosphate-Buffered Saline (PBS; composed of 0.32 g sodium dihydrogen phosphate and 1.09 g disodium hydrogen phosphate, both from Scharlau Chemie S.A., and 9 g NaCl from Labbox Labware S.L., per liter of distilled water). The prepared suspensions were stored at 4 °C for up to 24 h prior to use.

### 2.2. Proteolytic and Lipolytic Activity

Proteolytic agar was used to evaluate the enzymatic activity of the 142 selected strains. This activity was evaluated with modifications of the method described by Zeng et al. [20] to adapt the specificity of the samples to plant-based proteins. Proteolytic agar was elaborated with 0.1% isolated pea protein (HSN, Granada, Spain), 0.25% yeat extract (Condalab), 0.1% glucose (Labkem, Barcelona, Spain), and 1.5% agar. It was inoculated with the different microorganisms and incubated at 25 °C for 72 h. The agar plates were stained with 0.05% (*w/v*) Coomassie Brilliant Blue R-250 (Fisher Scientific S.L., Madrid, Spain) prepared in a methanol (Fisher Scientific S.L., Madrid, Spain): acetic acid (Panreac, Barcelona, Spain): distilled water solution (50:10:40, *v/v/v*). After 30 min, the plates were destained using a solution composed of methanol: ethanol (Scharlau Chemie S.A.): acetic acid:distilled water (20:10:5:65, *v/v/v/v*). Proteolytic activity was evidenced by the formation of halos. Lipolytic activity was also evaluated to rule out the strains with the highest activity, to prevent excessive oxidation during fermentation. Lipolytic agar was elaborated with nutrient broth (Conda Pronadisa), 1.5% agar (Conda Pronadisa), and 1% triolein (Sigma Aldrich Co., St. Louis, MO, USA). It was inoculated with the same 142 selected strains, and incubated at 25 °C for 10 days, with halos evaluated every 24 h for 7 days.

### 2.3. Identification of Selected Microorganisms by Whole Genomic Sequencing (WGS)

Those strains showing proteolytic activity and no relevant lipolytic activity were identified through WGS. For this, genomic DNA was isolated from overnight bacterial cultures following enzymatic cell lysis and subsequent purification using the DNeasy Blood & Tissue Kit (QIAGEN^®^, Ref. 69506, Hilden, Germany). The concentration and purity of the extracted DNA were determined fluorometrically using a Qubit 3.0 fluorometer and the dsDNA BR Assay Kit (Ref. Q32853). For WGS, barcoded libraries were generated with the Rapid Barcoding Kit 24 V14 (Oxford Nanopore Technologies, Oxford, UK; Ref. SQK-RBK-114.24), following the supplier’s recommended procedure. The prepared libraries were sequenced using R10 flow cells (Ref. FLOW-MIN-114) on a MinION Mk1D sequencing platform (Oxford Nanopore Technologies).

Base calling of the generated signal data was conducted with Dorado v7.11.2 using the high-accuracy model and a minimum quality threshold of Q9. Following base calling, FASTQ files corresponding to each strain were combined prior to downstream analysis. Low-quality and short sequences were subsequently removed using Filtlong v0.3.1. Reads shorter than 1000 bp were discarded, and the upper 90% of reads according to the Filtlong quality ranking were retained. Residual sequencing adapters were removed using Porechop_ABI v0.5.1 with the ab initio adapter detection option. The quality-filtered reads were subjected to de novo genome assembly through the Hybracter v0.11.2 workflow, which employed Flye v2.9.5-b1801 with the --nano-hq setting. Assembly consensus sequences were subsequently refined using Medaka v2.0.1 and the r1041_e82_400bps_sup_v5.0.0 model.

Genome-based taxonomic classification was initially performed with GTDB-Tk v2.6.1 (release r226). The resulting species assignments were subsequently cross-checked using FastANI v1.34 by comparing each assembly with its closest reference genome in the GTDB database.

### 2.4. Protein Fermentation

Isolated and texturized pea proteins were analyzed in the present study. Isolated pea protein (HSN) was mixed with glucose and distilled water (1.176:0.05:50; *w/w/w*). Texturized pea protein (Dacsa, Valencia, Spain) was mixed with glucose and distilled water (1.724:0.05:50; *w/w/w*). These mixtures were prepared to achieve a final 2% protein concentration each. This concentration was verified using the Bradford assay [21].

Proteins were inoculated with the selected proteolytic strains (GC 13, 15, 28, 40) in sterile centrifuge tubes at an approximate concentration of 5 log CFU/g and incubated at 25 °C for 24 and 48 h. An uninoculated control was also incubated. Every treatment was performed in triplicate.

### 2.5. Microbiological Analyses

Microbiological analyses were conducted for each elaboration on days 0, 1, and 2. Samples of approximately 1 g were taken and homogenized in Stomacher homogenizer bags (Interscience, Saint-Germain-en-Laye, France) with 9 mL of 1% (*w/v*) peptone water (Conda Pronadisa), followed by the preparation of serial decimal dilutions. Appropriate agar media were used for enumeration depending on the target microbial group. Plate Count Agar (PCA; Conda Pronadisa), Mannitol Salt Agar (MSA; Conda Pronadisa), Violet Red Bile Glucose agar (VRBG; Conda Pronadisa), and MRS agar were used for quantification of total viable counts, GC, enterobacteria, and LAB, respectively. PCA, MSA, and MRS were incubated at 30 °C for 48 h. VRBG was incubated at 37 °C for 24 h. Only plates with 10 to 300 colonies were considered for counting, and results were expressed as CFU/g.

### 2.6. Physicochemical Analyses

The analyses were conducted in triplicate in accordance with the guidelines established by the Association of Official Analytical Chemists [22]. A pH meter (Hach Lange Spain S.L.U., Barcelona, Spain) was employed to measure pH, which had been previously calibrated using three standard solutions (Scharlau Chemie S.A.). Water activity (a_w_) was determined at 25 °C with a specialized analyzer (Novasina AG, Lachen, Switzerland), utilizing standard salt solutions with known a_w_ values for calibration.

### 2.7. Free Amino Acids and Biogenic Amines Assessment

The determination of free amino acids (FAA) and biogenic amines was carried out following an adapted method described by [23]. Approximately 3–4 g of broth were homogenized with 10 mL of 0.6 M perchloric acid (Scharlau Chemie S.A.). The homogenate was centrifuged at 366.52 rad/s for 10 min at 4 °C (Eppendorf AG, Hamburg, Germany). The supernatant was transferred to clean polystyrene centrifuge tubes, and the pellet was washed with an equal volume of 0.6 M perchloric acid. The new mixture was centrifuged again under the same conditions, and the resulting supernatant was combined with the first one. The pooled extract was brought to pH 6.0 with potassium hydroxide (Scharlau Chemie S.A.), transferred to a 25 mL volumetric flask, and filled to volume with deionized water. Samples were subjected to two freeze–thaw cycles, and the clarified supernatant was collected after centrifugation. Immediately before High-Performance Liquid Chromatography (HPLC) analysis, samples were filtered through 0.22 μm membrane filters (RephiLe Bioscience, Philadelphia, PA, USA). All experiments were performed in triplicate for each substrate (isolated and texturized pea protein).

Chromatographic analysis was conducted under common conditions for both FAA and biogenic amine determinations. An aliquot of 8 µL of each sample was injected into a Dionex Ultimate 3000 UHPLC system (Thermo Fisher Scientific) equipped with a degassing module, a quaternary pump, an autosampler, and a thermostated (45 °C) Thermo Fisher Accucore Aq C18 column (150 × 2.1 mm, 2.6 μm particle size). The mobile phases consisted of HPLC-grade solvents: phase A (H_2_O with 0.1% formic acid) and phase B (acetonitrile with 0.1% formic acid). The flow rate was 0.4 mL/min, with a gradient from 2 to 45% B in 8 min, followed by 45–90% B in 5 min, and a 1 min wash at 90% B before re-equilibration to 2% B for 1 min. Retention times of analytes are reported in Table 1.

Identification and quantification of FAAs and biogenic amines were performed using a Q Exactive Plus mass spectrometer (Thermo Fisher Scientific) operating in Full Scan mode with an electrospray ionization (ESI) source (HESI II, Thermo Fisher Scientific) in positive ion mode (ESI^+^). Source conditions were as follows: spray voltage 4 kV; sheath gas (N_2_ > 95%) 50; auxiliary gas (N_2_ > 95%) 25; capillary temperature 350 °C; S-lens RF level 55. The automatic gain control (AGC) target values was 1 × 10^6^, with a scan range of 50–300 *m/z* at a resolution of 70,000 full width at half maximum (FWHM). Data acquisition and processing were performed using FreeStyle™ software, version 1.8 (Thermo Fisher Scientific). Each analyte was identified by its retention time and exact mass, within a tolerance of ≤5 ppm.

Standards of amino acids and amines were prepared in 20% acetonitrile and 80% deionized water (*v/v*) containing 50 mM formic acid. All chemicals were of analytical grade. Standards used included histidine (His), threonine (Thr), aspartic acid (Asp), glutamic acid (Glu), asparagine (Asn), serine (Ser), glycine (Gly), arginine (Arg), alanine (Ala), tyrosine (Tyr), cystine (Cys), valine (Val), methionine (Met), tryptophan (Trp), phenylalanine (Phe), isoleucine (Ile), leucine (Leu), lysine (Lys), proline (Pro), ornithine (Orn), and glutamine (Gln) for FAAs, and histamine (Him), tyramine (Tym), tryptamine (Trm), cadaverine (Cad), putrescine (Put), spermine (Spm), and spermidine (Spd) for biogenic amines. Eighteen calibration points (0.05–10 ng/mL) were prepared for each FAA, and sixteen points (0.0003–10 ng/mL) for each amine, both yielding linear calibration curves (r^2^ ≥ 0.99). Detection and quantification limits (LOD and LOQ), defined as signal-to-noise ratios of 3.5 and 10, respectively, are presented in Table 1.

### 2.8. Volatile Compounds Analysis

The analysis of volatile organic compounds (VOCs) was carried out in triplicate for each experimental group using solid-phase microextraction (SPME). For each assay, 3 g of sample were placed in 20 mL glass vials and equilibrated by heating at 40 °C for 25 min prior to extraction. Volatiles were subsequently adsorbed onto a 50/30 µm divinylbenzene/carboxen/polydimethylsiloxane (DVB/CAR/PDMS) fiber (Merck, Darmstadt, Germany). After extraction, analytes were thermally desorbed and analyzed by gas chromatography coupled to a mass spectrometer (GC–MS).

The analytical system consisted of an Agilent 6890 gas chromatograph (Santa Clara, CA, USA) equipped with a DB-1 capillary column (60 m × 0.25 mm × 1.2 µm) and interfaced with an Agilent 5975C mass selective detector. The GC oven temperature program was as follows: initial temperature of 40 °C (5 min hold), increased at 5 °C/min to 230 °C, then ramped at 25 °C/min to a final temperature of 250 °C. Volatile desorption from the SPME fiber was performed at 250 °C for 15 min, with the transfer line maintained at 280 °C. Helium (Air Liquide, Madrid, Spain) served as the carrier gas at a constant flow of 1.2 mL/min. Mass spectrometric detection operated in full-scan mode over the 50–350 amu mass range. Chromatographic peaks were manually integrated.

Compound identification was performed by comparing the mass spectra with reference data from the NIST/EPA/NIH library (National Institute of Standards and Technology, Gaithersburg, MD, USA). Linear retention indices (LRIs) were also calculated and confirmed by comparison with those obtained from a standard C6–C20 n-alkane mixture (Sigma Aldrich Co.) analyzed under identical chromatographic conditions. All analyses were conducted in triplicate for both isolated and texturized pea protein.

### 2.9. Statistical Analyses

Statistical distribution was assessed by means of the Shapiro–Wilk test, and homogeneity of variances was evaluated using Levene’s test. Depending on the results obtained, group comparisons were conducted employing either Tukey’s HSD, Kruskal–Wallis H, or Dunnett’s T3 procedures (*p* < 0.05). All statistical analyses were executed utilizing IBM SPSS Statistics, version 22 (IBM Co., New York, NY, USA).

Peak area data corresponding to VOCs were processed using RStudio software (2026.01.0). Relatively normalized volatile profiles and compound grouping were visualized via a hierarchical heatmap. Hierarchical clustering was performed on compounds using Euclidean distance as the dissimilarity metric and Ward’s minimum variance method as the agglomeration link.

## 3. Results

### 3.1. Selection of Microorganisms

Microorganisms were selected based on the proteolytic and lipolytic activities shown in the specific agars (Figure 1). GC strains 13, 15, 28, and 40 were the only microorganisms among the initial 142 that exhibited confirmed proteolytic activity within 72 h of incubation or less at 25 °C. Specifically, GC 13 and 28 formed halos at 48 h, while GC 15 and 40 did so at 72 h. GC strains 13 and 28 also exhibited low lipolytic activity within 6 days of incubation at 25 °C. The remaining microorganisms did not show lipolytic activity within the 7 days of incubation evaluated.

### 3.2. Identification of Selected Microorganisms by WGS

The bacterial isolates (GC 13, GC 15, GC 28, and 40), selected by for their proteolytic activity and their low or no lipolytic activity, were identified by WGS as *Staphylococcus warneri* (GC 13 and 28), *Rothia uropygioeca* (GC 15), and *Enterococcus lactis* (GC 40).

### 3.3. Physicochemical and Microbiological Assay

The inoculation of the four selected strains in pea protein isolate and textured pea protein led to measurable changes in both physicochemical and microbiological parameters over the 2-day fermentation period at 25 °C (Table 2). In both substrates, inoculated samples showed a gradual decrease in pH from the initial value of around 7.1. On the other hand, a_w_ remained around 0.980 with no significant differences during the fermentation period, or between the microorganisms or matrices tested. Generally, fermentation induced a more pronounced acidification between days 0 and 1 and smaller additional reductions by day 2. Uninoculated controls were also acidified, achieving a pH range similar to the inoculated matrices.

In pea protein isolate, inoculation with the four selected strains led to a moderate but significant acidification over time, with pH values decreasing from 7.15 at day 0 to 6.72–6.97 by day 1, and to 6.37–6.55 by day 2, depending on the strain (*p* < 0.05). Within each sampling day, no relevant strain-dependent differences in pH were evident, but significant reductions were observed across days for each strain, indicating active metabolic activity during fermentation. Total aerobic counts on PCA in inoculated samples increased from 4.84 to 5.88 log CFU/g at day 0, to 6.64–7.88 at day 1, and 7.15–7.49 log CFU/g at day 2 (*p* < 0.05). The uninoculated control also showed a significant increase (*p* < 0.05), reaching 7.83 log CFU/g counts at the end of the incubation period, up from <2 log CFU/g on the initial day. In the MSA, the inoculated experimental samples also displayed higher counts on the initial days (*p* < 0.05), ranging from 5.58 to 6.03 log CFU/g. GC 15 and 40 reached the highest concentration during fermentation, around 8.0 log CFU/g, which was significantly higher than those of GC 13 and 28 at day 1 and 2 of fermentation (*p* < 0.05). This fact highlighted strain-dependent growth in this matrix. Lactic acid bacteria enumerated on MRS remained below the detection limit at day 0 and 1 of fermentation in every sample, but by day 2, all inoculated samples, as well as the control, showed detectable populations of 3.35–4.56 log CFU/g.

In textured pea protein, the acidification effect was more pronounced than in protein isolate (*p* < 0.05), with pH decreasing from 7.08 at day 0 to 5.22–5.41 at day 2 for all samples (*p* < 0.05). Significant differences were detected between days for each strain, confirming a progressive decrease in pH irrespective of the inoculum (*p* < 0.05). Aerobic mesophilic counts on PCA rose from 5.06 to 5.52 log CFU/g at day 0 to 7.70–8.61 log CFU/g at day 2 (*p* < 0.05) in the inoculated samples. The uninoculated control only displayed lower counts than the inoculated samples on the initial day (*p* < 0.05), with 2.26 log CFU/g. During fermentation time, the aerobic counts in the uninoculated samples were similar to those inoculated with *S. warneri* GC 13 and GC 28 and *R. uropygioeca* GC 15 (*p* < 0.05). On MSA, batches inoculated with *R. uropygioeca* GC 15 and *E. lactis* GC 40 again reached the greatest final populations, around 8.5 log CFU/g, which were significantly higher than those of GC 13 and 28 at day 2 (*p* < 0.05), mirroring the pattern observed in the protein isolate. Counts on MRS significantly increased (*p* < 0.05) from values below the detection limit at day 0 in every sample to 6.30–8.30 log CFU/g at day 1 of fermentation and 7.33–8.56 log CFU/g at day 2. The uninoculated samples achieved the highest LAB counts on the first fermentation day, while a significant increase took place (*p* < 0.05) between the first and second fermentation days in the inoculated samples.

When comparing protein matrices, textured pea protein often supported higher microbial counts resulting from contamination than pea protein isolate, particularly for aerobic mesophiles and LAB (*p* < 0.05). For example, at day 1, the protein isolate and texturized protein uninoculated samples displayed 3.22 and 7.59 log CFU/g on PCA (*p* < 0.05), respectively. Likewise, MRS counts for all strains at days 1 and 2 were significantly higher in the textured protein than in the isolate (*p* < 0.05). Overall, time, strain, and the protein matrix all contributed significantly to the observed variability in pH and microbial growth, with *R. uropygioeca* GC 15 and *S. warneri* GC 28 showing the highest microbial performance and the lowest contamination levels across conditions.

### 3.4. Free Amino Acids

The free amino acids quantified >LOD are displayed in Figure 2. The initial free amino acid profile in protein isolate was characterized by low overall concentrations, with Tyr (4.07 µg/g), Ile/Leu (0.78 µg/g), Phe (0.57 µg/g), Gln (0.42 µg/g), and Val (0.31 µg/g) as the main detectable compounds, while other amino acids were detected below LOQ. Over fermentation time, some strains, particularly *S. warneri* GC 13 and *R. uropygioeca* GC 15, promoted an increase in several FAA (*p* < 0.05) from 7.02 µg/g to 60.90 and 38.83 µg/g, respectively, at day 2. Uninoculated samples also increased during fermentation (*p* < 0.05), achieving 41.83 µg/g on the second fermentation day. This increase was led by the rise of Tyr, Val, Phe, and Ile/Leu (*p* < 0.05). Differences among strains on a given sampling day were limited to the second day of fermentation, with GC 13, 15, and the uninoculated samples showing the highest FAA (*p* < 0.05).

In textured pea protein, the baseline FAA pattern was more diversified and generally higher than in the isolate, again with Tyr as the predominant amino acid and relevant contributions from Glu, Arg, Val, Phe, and Ile/Leu. Fermentation of the selected strains resulted in a pronounced accumulation of multiple FAA from the initial day, with higher concentrations by day 2 in samples fermented with GC 15, 28, and 40 (*p* < 0.05). Statistical analysis also revealed strain-dependent differences in this matrix, with GC 15 and 40 associated with higher levels of Asp, Arg, and Gln at day 2 than GC 13 and 28 (*p* < 0.05). When both matrices were compared, textured protein generally exhibited significantly higher contents of Glu, Val, Ile/Leu, Lys, Gln, and overall summation than protein isolate (*p* < 0.05) during fermentation. Tyr was the only amino acid with a higher concentration in the protein isolate than the textured protein (*p* < 0.05).

### 3.5. Biogenic Amines

The biogenic amines detected >LOD are displayed in Figure 3. The only biogenic amines quantified in the protein isolate were tyramine and tryptamine, and no histamine, cadaverine, putrescine, spermine, or spermidine were detected in any strain or control throughout the experiment. Tyramine concentrations remained close to 0.6 ng/g at day 0 and showed no statistically significant differences among strains, with only a slight, non-systematic increase for some treatments at days 1 and 2, so that total biogenic amine content stayed below 1.2 ng/g in all cases.

In textured pea protein, tyramine was again the predominant amine but reached substantially higher levels over time. By day 2, tyramine detected concentration was 162–212 ng/g in inoculated samples and around 348 ng/g in the uninoculated control. Total biogenic amines obtained significant increases over baseline values in the batches inoculated with *S. warneri* GC 28 and *E. lactis* 40, and in the uninoculated control batch (*p* < 0.05). It was identified that there was significantly higher total biogenic amine content in the textured matrix than in the isolate for each sample on the second fermentation day (*p* < 0.05).

### 3.6. Volatile Compounds

Volatile compounds were grouped into clusters to reflect their origin and evolution during fermentation of the two pea protein matrices (Figure 4). This clustering separates native volatiles present in the raw material at day 0 from those generated during the fermentation of pea protein isolate or textured pea protein. Volatile compound concentrations are displayed in the Supplementary Table S1.

The first cluster comprised volatiles already present in the raw pea ingredients at day 0, mainly aldehydes (pentanal, hexanal, heptanal, benzaldehyde, 2octenal, nonanal), together with 1octanol, 2pentylfuran, 2,2,4,6,6pentamethylheptane, 3octen2one and 2nonanone. During fermentation, several of these compounds decreased significantly over time (*p* < 0.05). For example, hexanal in the protein isolate decreased from about 21.7 AU × 10^6^ at day 0 in all lots to below 1 AU × 10^6^ at day 1 for inoculated batches, while remaining at 17.3 AU × 10^6^ in the uninoculated control (GC 0). At day 1, hexanal also differed significantly among strains and matrices. In the protein isolate, inoculated strains 0.37–0.93 AU × 10^6^, while the textured matrix showed lower residual hexanal, 0.05–0.38 AU × 10^6^ (*p* < 0.05). Other Cluster1 aldehydes showed similar, compound-specific patterns. For example, pentanal in the protein isolate started at about 0.93 × 10^6^ AU × 10^6^ for all lots and became nondetectable after fermentation in inoculated batches (*p* < 0.05), whereas the control at day 1 still contained 0.85 × 10^6^ AU × 10^6^. Heptanal in the protein isolate remained detectable only in GC 28 and GC 0 at day 1 (0.07 and 0.53 AU × 10^6^, respectively), while the same compound disappeared completely in textured pea protein after fermentation, underlining a matrix-specific attenuation of this aldehyde (*p* < 0.05).

Compounds in cluster 2 were generated during fermentation, including ramified aldehydes (2-methylbutanal, 2-ethyl-2-butenal, 2-butyl-2-octenal), higher alcohols (1-penten-3-ol, 1-pentanol, 1-hexanol), hydrocarbons, ketones and 2,4-bis(1,1-dimethylethyl)phenol. 1-Pentanol rose from 0.41 AU × 10^6^ at day 0 in all isolate lots to 0.62–0.69 AU × 10^6^ at day 1 for inoculated batches GC 13, 15 and 40, whereas the uninoculated control remained at 0.43 AU × 10^6^ (*p* < 0.05). Comparing matrices, at day 1, the protein isolate showed higher alcohol values, as 1-pentanol (0.43–0.69 AU × 10^6^), whereas textured pea protein only reached 0.08–0.18 AU × 10^6^ (*p* < 0.05). Moreover, alcohol levels increased through time, as can be observed in 1-hexanol, rising in the protein isolate from 0.53 AU × 10^6^ at day 0 to 4.49 and 4.94 AU × 10^6^ at day 1 for GC 15 and 40, respectively (*p* < 0.05), whereas GC 0 remained around 0.97 AU × 10^6^. In textured pea protein, 1-hexanol rose from 0.16 AU × 10^6^ at day 0 to values around 2 AU × 10^6^ in GC 15 and 40 (*p* < 0.05). Other compounds, such as ketones and phenols, were also produced during fermentation, as observed with 2-hexanone and 2,4-bis(1,1-dimethylethyl)phenol, which were not detected at day 0.

Cluster 3 includes volatiles that were formed or markedly enhanced in textured pea protein, especially short-chain acids and oxygenated ketones. Acetic acid, for instance, was not detected at day 0 in either matrix but appeared at day 1 in the textured product with values of 0.41 and 0.63 AU × 10^6^ for GC 13 and 0, respectively, while GC 15 and 28 showed only 0.05 and 0.25 AU × 10^6^, respectively. GC 15 displayed a significant reduction in comparison with GC 0 (*p* < 0.05). In pea protein isolate, acetic acid remained non-detectable over the whole period, so the presence of significant amounts in textured pea protein reflects a strong matrix effect (*p* < 0.05).

Among alcohols identified in cluster 3, 3-methyl1butanol or 1-octen-3-ol in textured pea protein increased over the fermentation time (*p* < 0.05). Ketones such as 2,3-butanedione, were also detected in the one-day fermented textured matrix, with 0.06 AU × 10^6^ in GC 13 and 0.13 AU × 10^6^ in GC 0 (*p* < 0.05). Similarly, 3-hydroxibutanone increased to 0.61 AU × 10^6^ in GC 13 and 1.05 AU × 10^6^ in the control at day 1 in textured pea protein, but was not detected in pea protein isolate, confirming a matrix specific effect (*p* < 0.05).

## 4. Discussion

### 4.1. Strain Selection

The screening of 142 microorganisms by proteolytic activity resulted in the detection of four proteolytic bacteria. These bacteria showed low or no detectable lipolytic activity. The four strains were finally characterized at species level by WGS as *S. warneri* (GC 13 and 28), *R. uropygioeca* (GC 15), and *E. lactis* (GC 40). The WGS methodology has become the gold standard for identifying bacteria species [24]. In addition, this methodology provides a full genetic blueprint of the strains tested, allowing to detect antimicrobial resistance genes and virulence factors after further analysis. Preliminary analysis of the WGS results of the strains *S. warneri* GC 13 and GC 28, *E. lactis* GC 40 and *R. uropygioeca* GC 15 showed that these strains do not harbour the main antibiotic resistance genes, including *vanA*/*vanB* resistence genes, nor the main virulence genes, including *esp*, *hyl*, *asa1*, *gelE* and *cylA*.

When assessing the ability of these strains to produce biogenic amines, their production levels in both isolated and textured pea protein are negligible. However, the tyramine levels detected in textured protein were 162–212 ng/g in inoculated samples, reaching 348 ng/g in uninoculated controls. The European Food Safety Authority (EFSA) considers tyramine safe at doses up to 600 mg per meal for healthy individuals without monoamine oxidase inhibitor (MAOI) medication but recommends levels below detectable limits for individuals with histamine intolerance [25]. The concentrations observed in this study, while elevated relative to unfermented plant proteins, remain within accepted thresholds for the general population and are substantially lower than typical levels in cheeses or cured sausages [26,27,28]. From a commercial product development perspective, *R. uropygioeca* GC 15 and *E. lactis* GC 40 could be positioned as contributing to the inhibition of biogenic amine formation by contaminating microbiota rather than as amine producers themselves. Although it cannot be conclusively determined whether the observed amines originate from the inoculated microorganisms or from associated microbiota, certain strains appear to reduce the overall amine-producing potential of the microbial community. Notably, variability was observed among the inoculated microorganisms, with some exhibiting a greater inhibitory capacity than others. Accordingly, the targeted selection of strains showing the strongest inhibitory effect on biogenic amine formation would represent a rational strategy for product optimization, fully consistent with EFSA recommendations regarding the control of biogenic amines in fermented foods.

### 4.2. Strain-Dependent and Matrix-Dependent Proteolytic Capacity

*Staphylococcus* sp. and *Rothia* sp., have already well-characterized serine proteases and general peptidases that collectively degrade casein, myofibrillar proteins, and plant storage proteins into oligopeptides and free amino acids [29,30]. Differences between similar strains in their capacity to liberate free amino acids have been found by other authors [31]. In the present study, *S. warneri* GC 13-fermented pea protein isolate accumulated 60.90 µg/g total FAA at day 2, compared to 14.59 µg/g in *S. warneri* GC 28-fermented protein isolate (*p* < 0.05), despite having proteolytic activity uniformly confirmed through agar-based assays. However, in the textured protein, *R. uropygioeca* GC 15, *S. warneri* GC 28, and *E. lactis* 40 were the only ones that showed a total FAA increase during fermentation (*p* < 0.05). Upon fermentation, *S. warneri* GC 13 and *R. uropygioeca* GC 15 promoted increases in multiple FAA (*p* < 0.05), suggesting that these strains expressed proteolytic enzymes with distinct substrate specificities and activity levels compared to *S. warneri* GC 28 and *E. lactis* 40. The differential proteolytic performance among strains likely reflects variability in protease gene expression, enzyme secretion efficiency, and peptidase complement, though these have not been evaluated in the present study. The evaluation of the complete genome, which will be performed in the future should provide information about these aspects.

The distinction between pea protein isolate and textured pea protein is reflected in the superior performance of all four strains in textured protein, wherein total FAA concentrations reached 9–64 µg/g by day 1 (*p* < 0.05), substantially exceeding the protein isolate values (1–26 µg/g). Other authors have evaluated how extrusion processing reduces the availability of free amino acids compared to the raw materials [32,33]. These findings suggest that the higher FAA concentration in the textured protein matrix might result from the diversity of nutrients that enhance microbial activity [34]. The presence of carbohydrates in textured protein can facilitate microbial fermentation and generate aromatic compounds [35,36]. Accordingly, the present work should be considered as a first screening step for the selection of promising microbial candidates, whereas peptide identification and functional validation constitute the next stage required to assess their full biotechnological potential.

While the present study focused on free amino acids as indicators of proteolytic activity, peptides generated during fermentation may also play an important role in determining the nutritional and functional properties of fermented protein matrices [15]. Previous studies have reported that microorganisms isolated from animal-derived fermented foods can release peptides exhibiting biological activities, including antioxidant, antihypertensive, antimicrobial, and immunomodulatory properties [17,18]. Therefore, the proteolytic potential of the strains investigated in this work could extend beyond the production of free amino acids. Nevertheless, the relationship between free amino acid accumulation and bioactive peptide production is complex. High concentrations of free amino acids may indicate extensive proteolysis but do not necessarily reflect the abundance of specific peptides, since these molecules can undergo further hydrolysis during fermentation. As a consequence, amino acid profiling alone does not provide direct information about the nature or functionality of the peptide fraction generated.

Therefore, future studies should include a detailed characterization of the fermentation peptidome as well as the microbial proteome to elucidate the enzymatic mechanisms involved in protein degradation and to identify potential bioactive peptides generated during fermentation of plant protein substrates.

### 4.3. Matrix-Dependent Acidification and Microbiological Dynamics

The differences observed in acidification and microbial growth between protein isolate and textured protein reflect fundamental differences in nutrient accessibility and the capacity of each matrix to support rapid heterotrophic microbial proliferation. This observation had already been studied in products elaborated with plant-based vs. animal-derived ingredients [37], though it is a novel finding across different plant-based processings. In pea protein isolate, despite the deliberate addition of 0.05% glucose to both matrices, the protein-dominant, carbohydrate-limited environment suppressed rapid acidification compared with textured protein (*p* < 0.05). In protein isolate, pH decreased by 0.18–0.43 on the first day of fermentation and 0.60–0.78 on the second day, indicating a substantial decrease over fermentation time (*p* < 0.05). Meanwhile, textured protein displayed 0.71–1.28 on the first day of fermentation and 1.67–1.86 on the second day, with a significant decrease over time (*p* < 0.05). LAB counts on MRS increased from <2 on the initial day on both proteins to 3.35–4.56 log CFU/g at day 25 in protein isolate and 6.30–8.30 log CFU/g in textured protein (*p* < 0.05). LAB are usually spoilage microorganisms responsible for acid production [38]. Moreover, they are traditionally defined by their ability to degrade carbohydrates [39,40], so this could explain why textured protein reached higher LAB counts and lower pH (*p* < 0.05). From a biocontrol perspective, the textured matrix would benefit from co-inoculation with defined LAB starter cultures to accelerate pH reduction and stabilize fermentation. Future studies might evaluate the combined effect of LAB and GC.

It has also been observed that *R. uropygioeca* GC 15 and *E. lactis* GC 40 achieved higher populations on MSA than GC 13 and 28 (*S. warneri*). These were also the strains that displayed a better biogenic amine safety profile, suggesting that strains *R. uropygioeca* GC 15 GC 15 and *E. lactis* GC 40 remain promising candidates for commercial development as single- or multi-strain starter cultures for plant protein fermentation.

### 4.4. Integration of Proteolysis, Acidification, and Strain Metabolism in Volatile Compounds Formation

The three-cluster organization of volatile compounds reflects the temporal and metabolic stratification of flavor development during fermentation: cluster 1 (native aldehydes and ketones) showed rapid depletion, cluster 2 (alcohols, nitrohydrocarbons, phenols) accumulated preferentially in isolate, and cluster 3 (acids, branched aldehydes, diketones) formed specifically in textured protein.

Cluster 1 contains several compounds usually related to beany off-flavor, such as pentanal, hexanal, heptenal, 2-octenal, and nonanal [9,41,42]. For example, hexanal concentration at day 0 was 21.7 × 10^6^ AU across all protein isolate samples, resulting in a reduction to <1.5 × 10^6^ AU by day 1 in inoculated strains vs. 17.29 × 10^6^ AU in uninoculated controls (*p* < 0.05). The removal of beany off-flavor is usually intended through chemical and physical methods, though biological strategies are emerging as a potential solution [43]. The aldehyde reduction achieved in the present study demonstrates that fermentation-specific microbial enzymatic activity drives the elimination of off-flavor compounds.

The transformation of aldehydes found in Cluster 1 to other compounds found in Cluster 2 and 3, such as 1-pentanol, 1-hexanol, and 2-heptanol, might provide evidence for aldehyde-to-alcohol conversion via alcohol dehydrogenase activity, as it has been evaluated by previous authors [44,45]. This enzymatic transformation has been found in fermented pulse protein by other authors [46,47], though it is not clearly positively defined as favorable from a sensory perspective [48,49].

Evaluating among-strain differences in aldehyde reduction, it was observed that GC 15 and 40 achieved lower concentrations, independent of the matrix evaluated (*p* < 0.05). Moreover, it is worth noting that *R. uropygioeca* GC 15 achieved the highest FAA concentration during fermentation in both protein matrices (*p* < 0.05), which could justify its use as a starter culture in pea protein fermentation.

Textured pea protein is especially formed by Cluster 3 compounds, with acids such as acetic acid, 3-methyl-butanoic acid, and 2-methyl-butanoic acid among them. According to other authors, these acids may result from LAB fermentation [50,51], which grew rapidly in textured protein. Though every inoculated textured protein sample displayed lower LAB values during fermentation (*p* < 0.05), only *R. uropygioeca* GC 15 showed a lower acetic acid concentration than the uninoculated control (*p* < 0.05). Thus, this fact is not related to the protective effect of GC 15 against LAB, but to the metabolic effect of the GC inoculation, which would be interesting to evaluate in future studies.

Diketones and hydroxyketones, such as 2,3-butanedione and 3-hydroxybutanone, contribute to buttery, creamy, and yogurt-like sensory notes, being typically found in LAB-fermented products [52]. Their dominance in textured protein reflects the higher LAB population density found in this matrix (*p* < 0.05). These compounds were not detected in the GC 15 fermented textured protein, which might be related to the metabolic effect of this GC, as observed with acetic acid. In contrast, 2,3-butanedione and 3-hydroxybutanone were absent from the evaluated protein isolate despite inoculation with the same strains, which underscores the critical importance of matrix-dependent metabolic adaptation.

The enhancement of protein quality through fermentation-driven proteolysis and free amino acid accumulation represents a significant nutritional advancement for plant-based protein products [53]. Plant proteins, particularly pea protein isolate, are constrained by lower digestibility scores relative to animal proteins, a limitation attributable to both limited essential amino acid content and reduced bioavailability due to anti-nutritive factors and limited pre-digestion by exogenous proteases [54]. Other authors are currently evaluating these challenges through different enzymatic treatments [55]. The present study increased the available amino acids, measured as free amino acids.

In addition to protein hydrolysis, microorganisms can simultaneously produce flavor-active compounds, organic acids, and other metabolites that contribute to sensory quality and product stability. Therefore, fermentation can combine protein modification and flavor generation within a single processing step, reducing the need for additional ingredients or processing aids, as it has also been evaluated by other authors [39,40]. Nevertheless, fermentation also presents certain limitations. Unlike isolated proteases, whose activity can be precisely dosed and controlled, microbial proteolysis depends on microbial growth, metabolic activity, substrate characteristics, and interactions with the resident microbiota [10]. The selection of suitable microbial strains is therefore critical to maximize these benefits and ensure a safe and consistent fermentation process.

Future research should incorporate transcriptomic and proteomic analysis to decipher the molecular mechanisms underlying strain-specific differences in proteolytic capacity, volatile formation, and biogenic amine production.

The selected strains belong to species not included in the Qualified Presumption of Safety (QPS) list of the European Food Safety Authority (EFSA), but certain strains of *S. warneri* and *E. lactis* have been considered as autochthonous functional starter cultures which can be used in industrial production, since they are natural microbial populations of ripened or fermented foods [56,57]. A further full genetic analysis of the WGS results of the four selected strains will allow evaluation of the safety of their use in foods, informing rational strain selection and potentially enabling targeted mutagenesis to eliminate undesirable traits while preserving beneficial ones. Furthermore, a sensory research will be conducted, integrating time-intensity flavor assessment, characterization of the fermented products, and consumer preference testing, which will establish the organoleptic relevance of the chemical changes documented in the present study and guide optimization of fermentation parameters toward commercially viable flavor profiles. Importantly, the rationale of the study was not to evaluate proteolysis under aseptic conditions, but rather to determine whether selected strains could be successfully applied to non-sterilized plant protein matrices. This represents a relevant industrial challenge because the sterilization of protein ingredients increases processing costs and may alter their functional properties. Our results suggest that the appropriate selection of starter cultures may enable the use of non-sterilized plant proteins while maintaining desirable fermentation performance and acceptable microbiological safety.

## 5. Conclusions

The screening and fermentation-driven metabolic profiling of four proteolytic bacteria (*S. warneri* GC 13, 28, *R. uropygioeca* GC 15, and *E. lactis* GC 40) demonstrates that controlled microbial fermentation can offer a clean-label approach to address flavor improvement and nutritional limitations of plant-based proteins while controlling biogenic amine production. The strain-dependent generation of free amino acids enhanced protein quality and bioavailability, while degrading native off-flavor aldehydes. The matrix-dependent dichotomy revealed how protein processing history determines fermentation outcomes and underscores the necessity for matrix-specific optimization of starter culture selection and fermentation parameters. From a practical biotechnology standpoint, *R.uropygioeca* GC 15 achieved higher free amino acid accumulation, favorable volatile profiles, and superior microbial growth kinetics while maintaining acceptable biogenic amine safety profiles. These findings demonstrate that selected microorganisms can be successfully applied to non-sterilized plant protein substrates, supporting the development of fermentation processes that may reduce or avoid the need for prior sterilization of theprotein ingredients while maintaining process safety. Further analysis by using whole-genome sequencing are needed to ensure the safety of the four selected strains before they are proposed for industrial use.

## Figures and Tables

**Figure 1:**
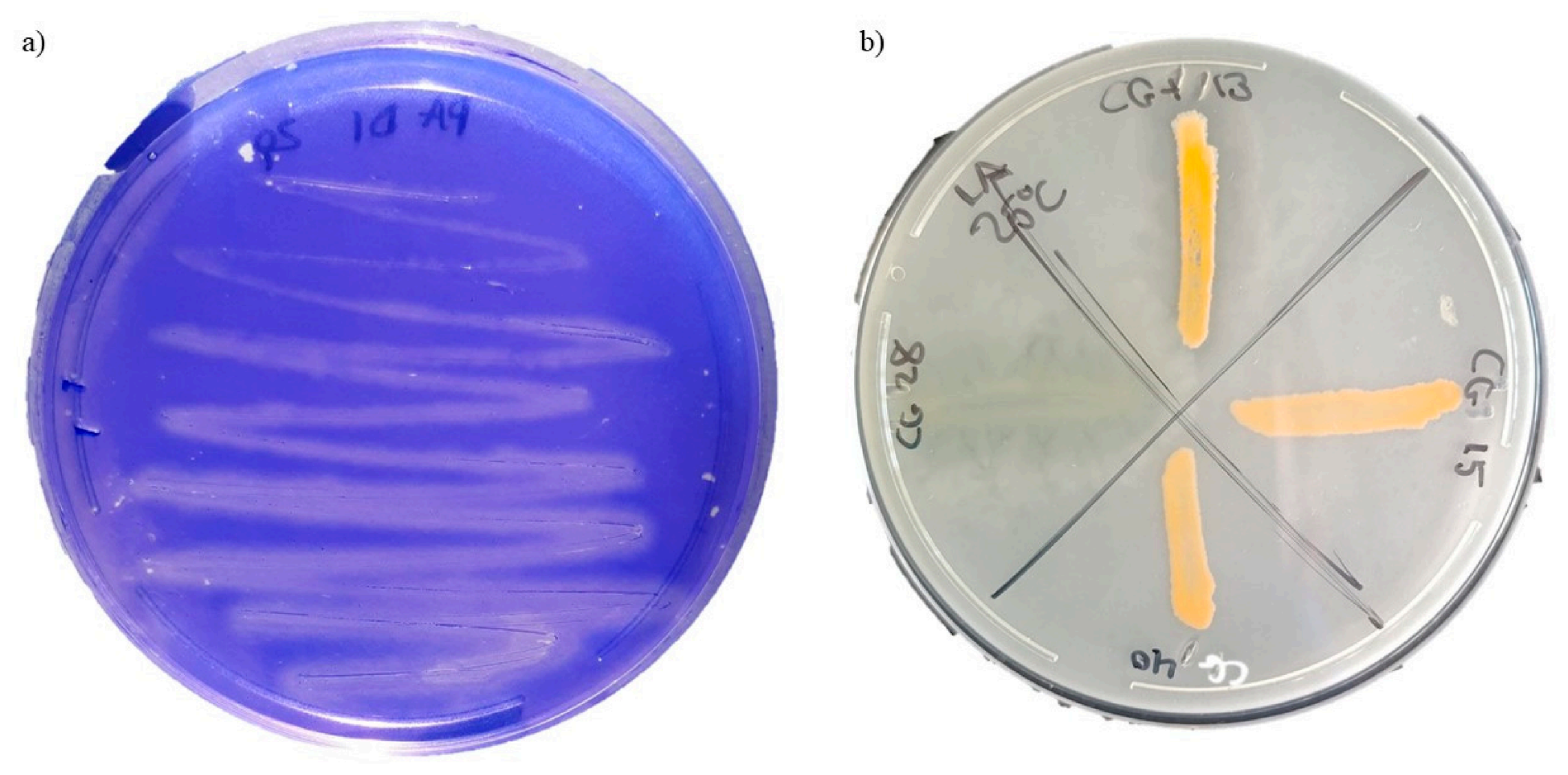
Proteolytic (**a**) and lipolytic (**b**) agar plates showing halo formation around colonies of positive microorganisms. Clear zones indicate enzymatic activity resulting from the hydrolysis of protein or lipid substrates after incubation at 25 °C.

**Figure 2:**
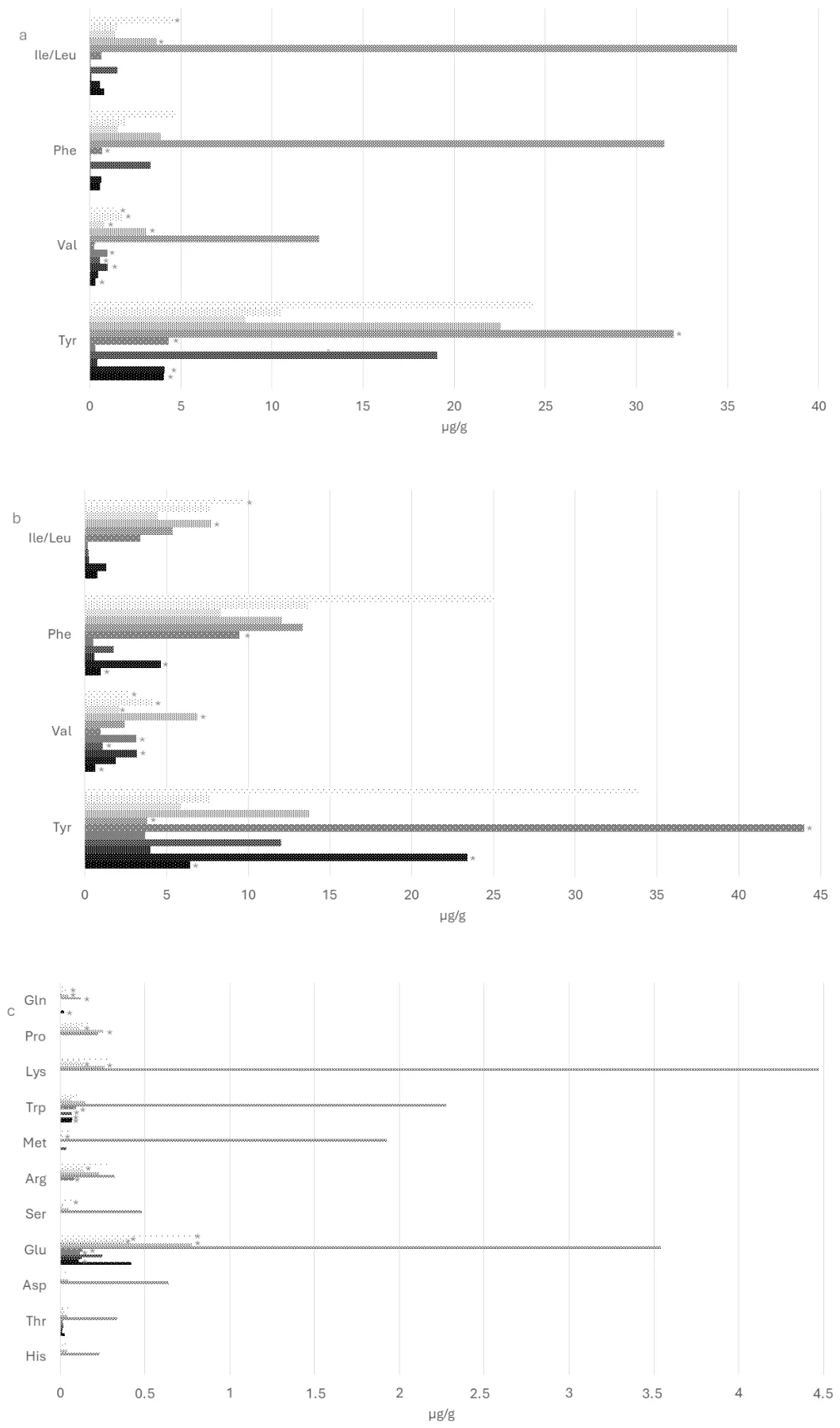

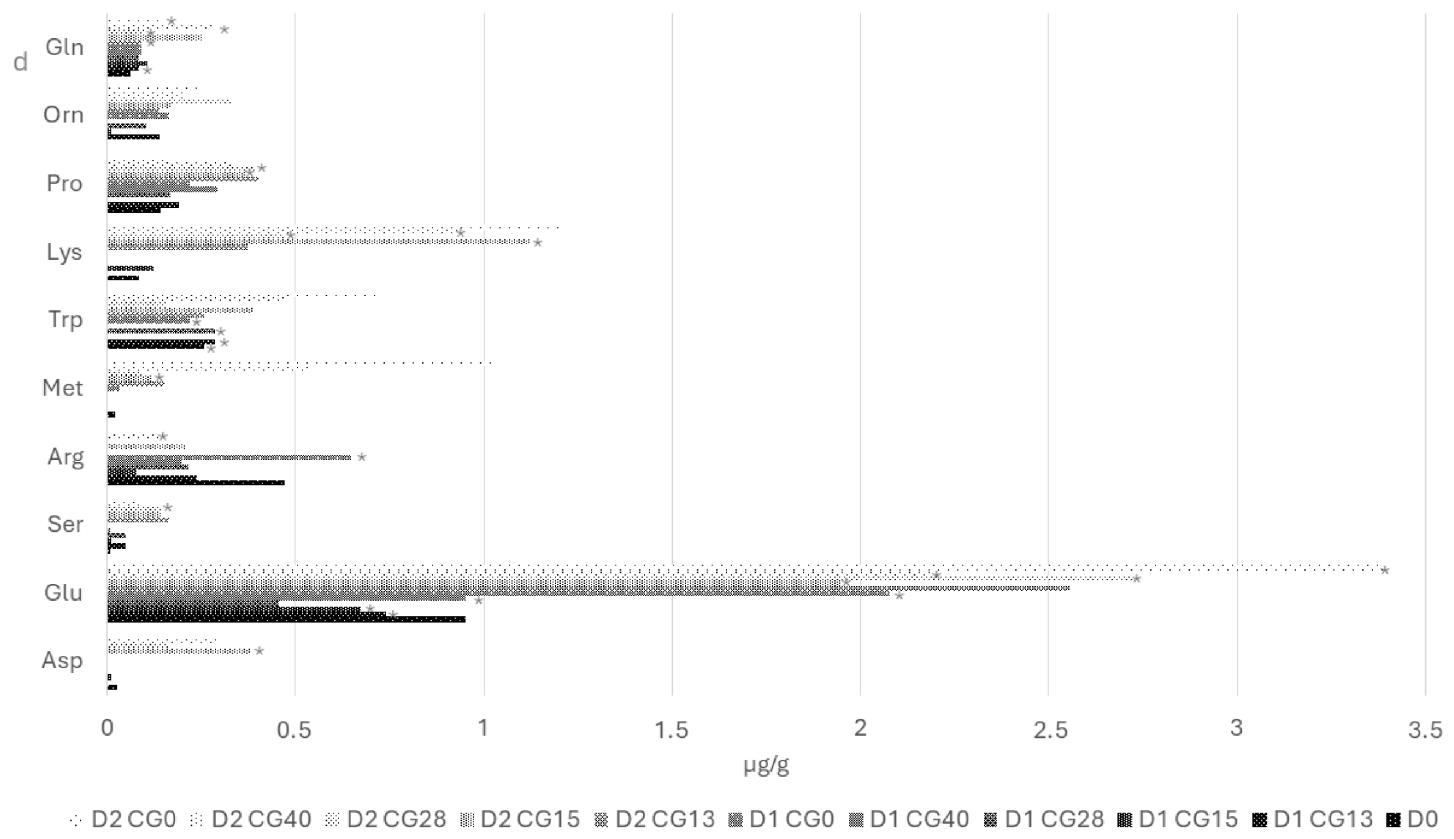
Free amino acids of the four selected strains (GC 13, 15, 28, and 40) and an uninoculated control (GC 0) inoculated into pea protein isolate (**a**,**c**) and textured pea protein (**b**,**d**) during fermentation (day 0, D0; day 1, D1; day 2, D2). Results are expressed as µg/g. The amino acids displayed were the ones quantified > LOD. Histidine (His), threonine (Thr), aspartic acid (Asp), glutamic acid (Glu), asparagine (Asn), serine (Ser), glycine (Gly), arginine (Arg), alanine (Ala), tyrosine (Tyr), cystine (Cys), valine (Val), methionine (Met), tryptophan (Trp), phenylalanine (Phe), isoleucine/leucine (Ile/Leu), lysine (Lys), proline (Pro), ornithine (Orn), and glutamine (Gln). Asterisks (*) denote differences between protein matrices for a given strain and sampling time (*p* < 0.05).

**Figure 3:**
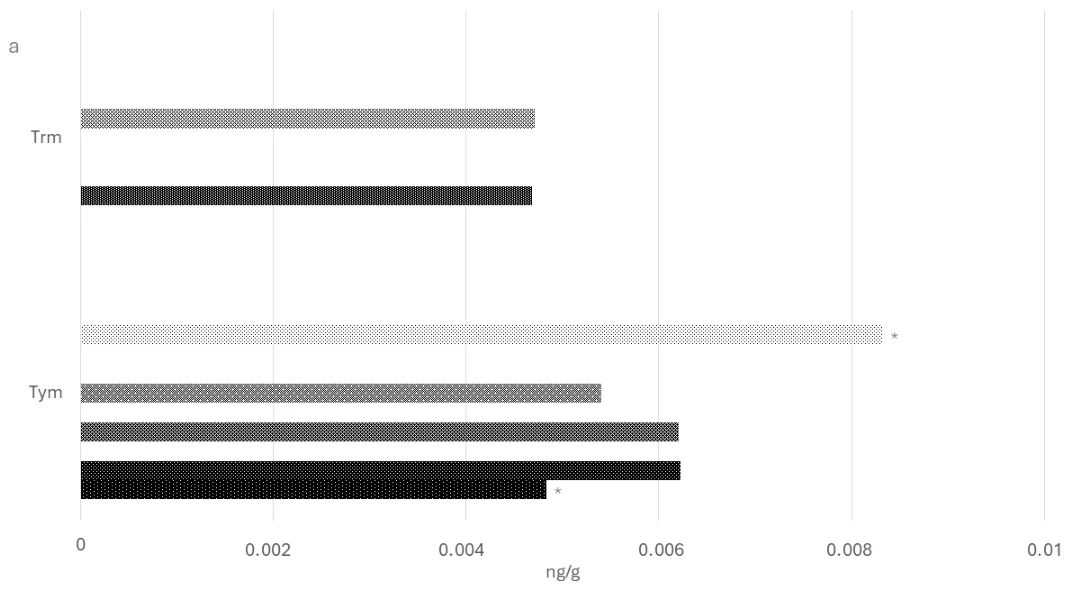

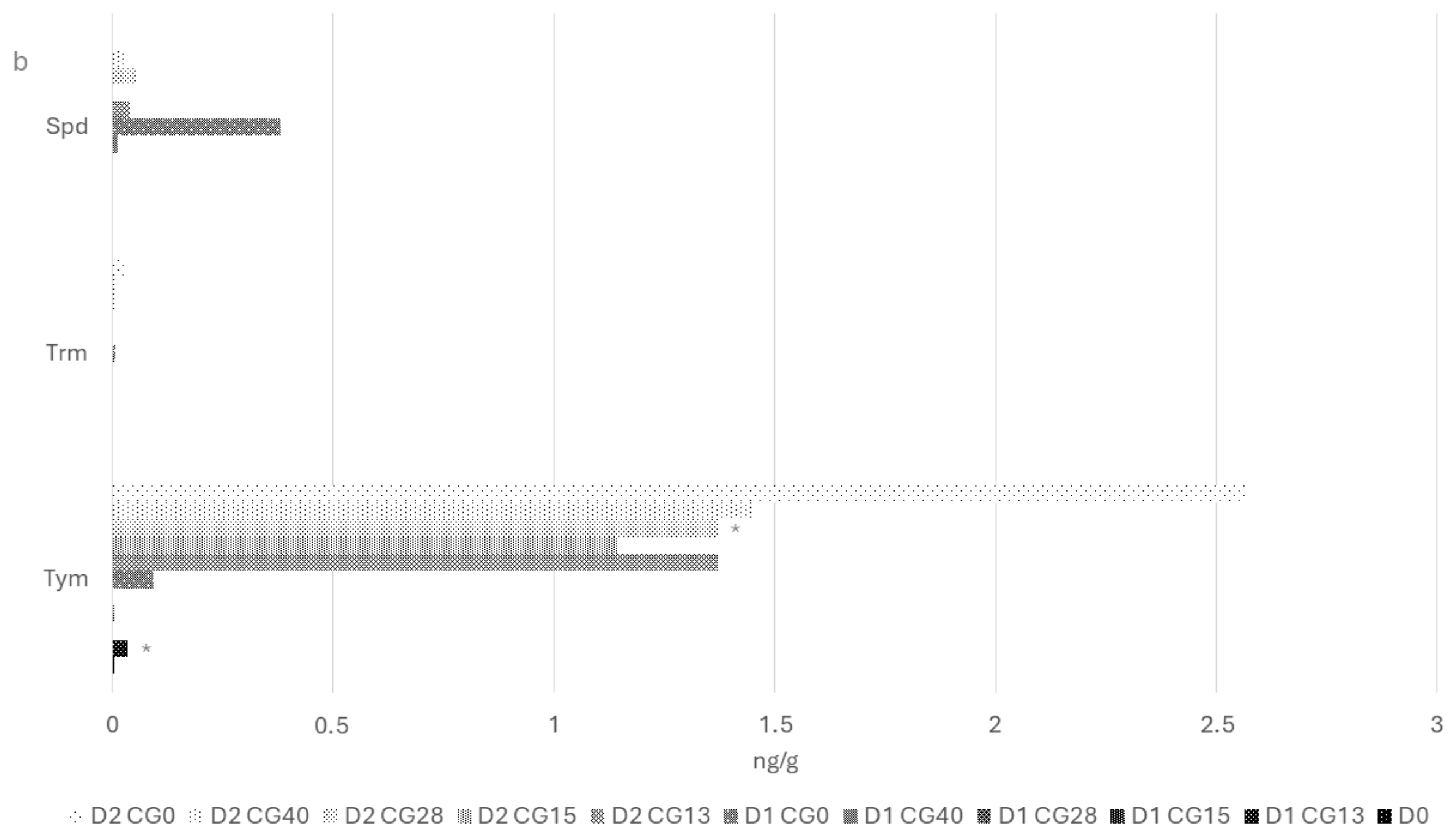
Biogenic amines evolution of the four selected strains (GC 13, 15, 28, and 40) and an uninoculated control (GC 0) inoculated into pea protein isolate (**a**) and textured pea protein (**b**) during fermentation (day 0, D0; day 1, D1; day 2, D2). Results are expressed as ng/g. The biogenic amines displayed were those quantified above the LOD. Spermidine (Spd), tryptamine (Trm), tyramine (Tym). Asterisks (*) denote differences between protein matrices for a given strain and sampling time (*p* < 0.05).

**Figure 4:**
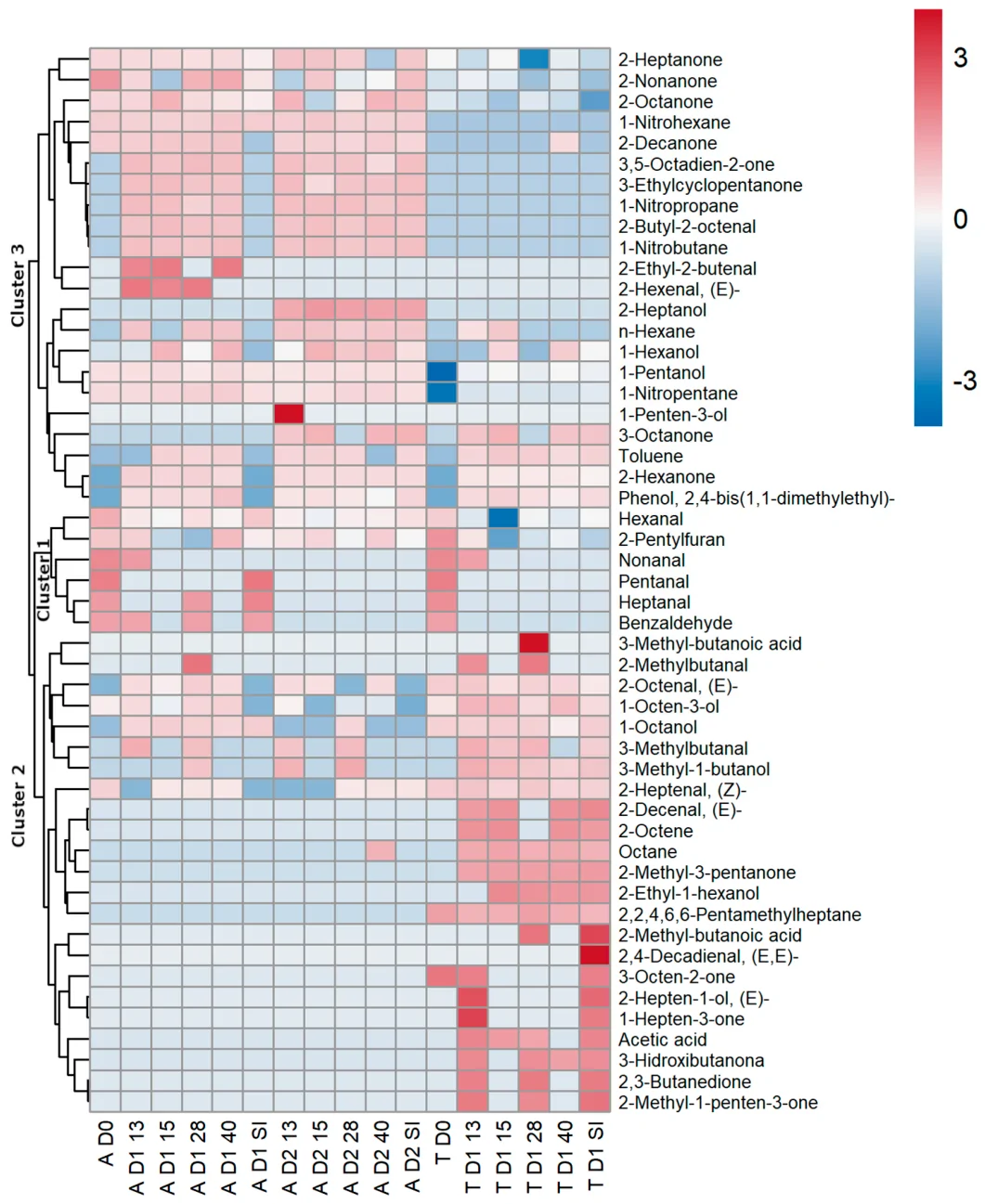
Heat map displaying clusters for the pea protein isolate (A) and textured pea protein (T) fermentation (day 0, D0; day 1, D1; day 2, D2) with the four selected strains (GC 13, 15, 28, and 40) and an uninoculated control (GC 0).

**Table 1:** Limit of detection (LOD), limit of quantification (LOQ) and retention time (RT; min) for the different free amino acids and biogenic amines sampled (µg/g).

Compound	LOD	LOQ	RT
Histidine	5.2 × 10^−3^	1.8 × 10^−2^	0.68
Threonine	5.2 × 10^−3^	1.8 × 10^−2^	0.70
Aspartic acid	5.2 × 10^−3^	1.8 × 10^−2^	0.70
Glutamic acid	5.2 × 10^−3^	1.8 × 10^−2^	0.70
Asparagine	5.2 × 10^−3^	1.8 × 10^−2^	0.68
Serine	5.2 × 10^−3^	1.8 × 10^−2^	0.68
Glycine	5.2 × 10^−3^	1.8 × 10^−2^	0.68
Arginine	5.2 × 10^−3^	1.8 × 10^−2^	0.72
Tyrosine	5.2 × 10^−3^	1.8 × 10^−2^	1.13
Cysteine	5.2 × 10^−3^	1.8 × 10^−2^	0.68
Valine	5.2 × 10^−3^	1.8 × 10^−2^	0.86
Methionine	5.2 × 10^−3^	1.8 × 10^−2^	1.01
Tryptophan	5.2 × 10^−3^	1.8 × 10^−2^	2.84
Phenylalanine	5.2 × 10^−3^	1.8 × 10^−2^	2.02
Isoleucine/Leucine	5.2 × 10^−3^	1.8 × 10^−2^	1.32
Lysine	5.2 × 10^−3^	1.8 × 10^−2^	0.70
Proline	5.2 × 10^−3^	1.8 × 10^−2^	0.74
Ornithine	5.2 × 10^−3^	1.8 × 10^−2^	0.70
Glutamine	5.2 × 10^−3^	1.8 × 10^−2^	0.68
Histamine	2.4 × 10^−3^	8.4 × 10^−3^	0.66
Tyramine	3 × 10^−4^	1 × 10^−3^	1.32
Tryptamine	2.4 × 10^−3^	8.4 × 10^−3^	3.37
Cadaverine	2.4 × 10^−3^	8.4 × 10^−3^	0.66
Putrescine	2.4 × 10^−3^	8.4 × 10^−3^	0.64
Spermine	3 × 10^−1^	1.05	0.62
Spermidine	3 × 10^−4^	1 × 10^−3^	0.64

**Table 2:** Microbial and pH evolution of the four selected strains (GC 13, 15, 28, and 40) and an uninoculated control (GC 0) inoculated into pea protein isolate and textured pea protein during fermentation.

Day	GC	pH	PCA (log CFU/g)	MSA (log CFU/g)	MRS (log CFU/g)
Pea protein isolate					
0	0	7.15 ± 0.05 ^a^	<2	<2	<2
	13	7.15 ± 0.05 ^a^	5.58 ± 0.04 ^h,i,j^	5.58 ± 0.10 ^i,j^	<2
	15	7.15 ± 0.05 ^a^	5.88 ± 0.04 ^g,h,i,j^	6.03 ± 0.10 ^h,i^	<2
	28	7.15 ± 0.05 ^a^	5.74 ± 0.06 ^h,i,j^	5.72 ± 0.12 ^i,j^	<2
	40	7.15 ± 0.05 ^a^	4.84 ± 0.17 ^a,1^	5.80 ± 0.07 ^i,j^	<2
1	0	6.97 ± 0.11 ^a,b^	3.22 ± 0.58 ^k^	<2	<2
	13	6.95 ± 0.02 ^a,b,c^	6.64 ± 0.29 ^e,f,g,h^	6.55 ± 0.17 ^g,h^	<2
	15	6.77 ± 0.02 ^a,b,c,d^	7.13 ± 0.93 ^d,e,f^	8.01 ± 0.12 ^a,b,c^	<2
	28	6.72 ± 0.47 ^a,b,c,d^	6.91 ± 0.14 ^d,e,f,g^	6.87 ± 0.25 ^f,g^	<2
	40	6.74 ± 0.02 ^a,b,c,d^	7.88 ± 0.34 ^a,b,c,d^	8.09 ± 0.21 ^a,b^	<2
2	0	6.52 ± 0.06 ^b,c,d^	7.83 ± 0.22 ^a,b,c,d^	<2	3.35 ± 0.38 ^g^
	13	6.43 ± 0.18 ^d,e^	7.35 ± 0.17 ^c,d,e,f^	6.98 ± 0.25 ^f,g^	4.56 ± 0.04 ^e^
	15	6.49 ± 0.10 ^c,d^	7.40 ± 0.55 ^c,d,e,f^	7.78 ± 0.22 ^b,c,d^	4.10 ± 0.11 ^e,f,g^
	28	6.37 ± 0.40 ^d,e^	7.49 ± 0.52 ^c,d,e^	7.22 ± 0.59 ^d,e,f,g^	3.63 ± 0.95 ^f,g^
	40	6.55 ± 0.06 ^b,c,d^	7.15 ± 0.37 ^d,e,f^	8.01 ± 0.13 ^a,b,c^	4.38 ± 0.19 ^e,f^
Texturized pea protein					
0	0	7.08 ± 0.03 ^a^	2.26 ± 0.25 ^k^	<2	<2
	13	7.08 ± 0.03 ^a^	5.43 ± 0.18 ^i,j^	5.46 ± 0.32 ^i,j^	<2
	15	7.08 ± 0.03 ^a^	5.52 ± 0.32 ^i,j^	5.87 ± 0.16 ^i,j^	<2
	28	7.08 ± 0.03 ^a^	5.16 ± 0.12 ^j^	5.30 ± 0.10 ^i^	<2
	40	7.08 ± 0.03 ^a^	5.06 ± 0.38 ^j^	5.57 ± 0.10 ^i,j^	<2
1	0	5.80 ± 0.18 ^f,g^	7.59 ± 0.07 ^b,c,d,e^	<2	8.30 ± 0.19 ^a^
	13	5.97 ± 0.06 ^e,f^	7.48 ± 0.22 ^c,d,e^	7.25 ± 0.29 ^d,e,f^	6.63 ± 0.16 ^c,d^
	15	6.34 ± 0.03 ^d,e^	7.50 ± 0.70 ^c,d,e^	7.76 ± 0.27 ^b,c,d^	6.53 ± 0.19 ^d^
	28	6.35 ± 0.17 ^d,e^	7.42 ± 0.09 ^c,d,e,f^	7.34 ± 0.33 ^c,d,e,f^	6.54 ± 0.18 ^c,d^
	40	6.37 ± 0.07 ^d,e^	6.36 ± 0.19 ^f,g,h,i^	7.79 ± 0.15 ^b,c,d^	6.30 ± 0.04 ^d^
2	0	5.41 ± 0.27 ^g,h^	8.70 ± 0.10 ^a^	<2	8.56 ± 0.14 ^a^
	13	5.22 ± 0.13 ^h^	7.78 ± 0.25 ^a,b,c,d^	7.05 ± 0.19 ^e,f,g^	7.45 ± 0.68 ^b^
	15	5.34 ± 0.14 ^g,h^	8.36 ± 0.27 ^a,b,c^	8.47 ± 0.25 ^a^	7.44 ± 0.26 ^b^
	28	5.29 ± 0.06 ^h^	7.70 ± 0.24 ^a,b,c,d,e^	7.69 ± 0.35 ^b,c,d,e^	7.33 ± 0.22 ^b,c^
	40	5.36 ± 0.07 ^g,h^	8.61 ± 0.18 ^a,b^	8.57 ± 0.14 ^a^	7.33 ± 0.09 ^b,c^

Results are expressed as mean values ± standard deviation. Microbiological results for total aerobic mesophiles (PCA), Gram-positive cocci (MSA), and lactic acid bacteria (MRS) are expressed as log CFU/g. Different superscript letters (a–k) denote significant differences between samples (*p* < 0.05).

## Data Availability

The original contributions presented in the study are included in the article. Further inquiries can be directed to the corresponding author. The complete genome sequences of GC 13, 15, 28 and 40 have been deposited at DDBJ/ENA/GenBank under the accession JCDQGV000000000, JCDQGU000000000, JCDQGT000000000, and JCEMDI000000000, respectively.

## Supplementary Materials

The following supporting information can be downloaded at: https://www.mdpi.com/article/10.3390/foods15213870/s1, Supplementary Table S1. Peak area count ±SD (×10^6^) of volatile compounds identified by HS-SPME/GC-MS in pea protein isolate (a) and textured pea protein (b) inoculated with the four selected strains (CG 13, 15, 28, and 40) and an uninoculated control (CG 0).

## Abbreviations

The following abbreviations are used in this manuscript:

GC	Gram-positive cocci
LAB	Lactic acid bacteria
FAA	Free amino acids
VOC	Volatile organic compound
