# Supplementary material for: Proteolytic Activity and Volatile Compound Formation by Staphylococcus warneri, Rothia uropygioeca, and Enterococcus lactis in Pea Protein Fermentations
Source: Foods. 2026 Oct 29;15(21):3870. doi: 10.3390/foods15213870 (PMC13763172; doi:10.3390/foods15213870)
Supplement: Supplementary file 1 [file foods-15-03870-s001.zip › Supplementary Table S1.pdf]

Supplementary Table S1. Peak area count ± SD (× 10^6) of volatile compounds identified by HS-SPME/GC-MS in pea protein isolate (a) and textured pea protein (b) inoculated with the four selected strains (CG 13, 15, 28, and 40) and an uninoculated control (CG 0).

a)

Name	id.	D0 CG 13	D0 CG 15	D0 CG 28	D0 CG 40	D0 CG0	D1 CG13	D1 CG15	D1 CG28	D1 CG40	D1 CG0	D2 CG13	D2 CG15	D2 CG28	D2 CG40	D2 CG0
Acids																
Acetic acid	MS	n.d.	n.d.	n.d.	n.d.	n.d.	n.d.	n.d.	n.d.	n.d.	n.d.	n.d.	n.d.	n.d.	n.d.	n.d.
3-Methyl-butanoic acid	MS	n.d.	n.d.	n.d.	n.d.	n.d.	n.d.	n.d.	n.d.	n.d.	n.d.	n.d.	n.d.	n.d.	n.d.	n.d.
2-Methyl-butanoic acid	MS	n.d.	n.d.	n.d.	n.d.	n.d.	n.d.	n.d.	n.d.	n.d.	n.d.	n.d.	n.d.	n.d.	n.d.	n.d.
Aldehydes																
3-Methylbutanal	MS, KI	n.d.	n.d.	n.d.	n.d.	n.d.	0.86 ± 0.06^{2}	n.d.	1.43 ± 0.32*	n.d.	n.d.	0.12 ± 0.03^{1}	n.d.	0.17 ± 0.11	n.d.	n.d.
2-Methylbutanal	MS, KI	n.d.	n.d.	n.d.	n.d.	n.d.	n.d.	n.d.	0.13 ± 0.00*	n.d.	n.d.	n.d.	n.d.	n.d.	n.d.	n.d.
Pentanal	MS, KI	0.93 ± 0.13*	0.93 ± 0.13*	0.93 ± 0.13*	0.93 ± 0.13*	0.93 ± 0.13*	n.d.	n.d.	n.d.	n.d.	0.85 ± 0.03	n.d.	n.d.	n.d.	n.d.	n.d.
Hexanal	MS, KI	21.67 ± 0.35^{2*}	21.67 ± 0.35^{3*}	21.67 ± 0.35^{2*}	21.67 ± 0.35^{3*}	21.67 ± 0.35^{2*}	0.93 ± 0.26^{ab1*}	0.37 ± 0.03^{a2}	1.38 ± 1.23^{ab12}	0.40 ± 0.04^{ab2*}	17.29 ± 0.43^{b12*}	0.29 ± 0.19^{1}	0.17 ± 0.03^{1}	0.23 ± 0.07^{1}	0.21 ± 0.01^{1}	0.53 ± 0.37^{1}
2-Ethyl-2-butenal	MS	n.d.	n.d.	n.d.	n.d.	n.d.	0.10 ± 0.01^{ab}	0.09 ± 0.00^{a}	n.d.	0.12 ± 0.01^{b}	n.d.	n.d.	n.d.	n.d.	n.d.	n.d.
2-Hexenal, (E)-	MS, KI	n.d.	n.d.	n.d.	n.d.	n.d.	0.09 ± 0.04	0.05 ± 0.00	0.07 ± 0.02	n.d.	n.d.	n.d.	n.d.	n.d.	n.d.	n.d.
Heptanal	MS, KI	0.82 ± [illegible]	0.82 ± [illegible]	0.82 ± [illegible]	0.82 ± [illegible]	0.82 ± 0.06^{2}	n.d.	n.d.	0.07 ± 0.02^{a1}	n.d.	0.53 ± 0.05^{b1}	n.d.	n.d.	n.d.	n.d.	n.d.

		0.06	0.06	0.06^{2}	0.06											
2-Heptenal, (Z)-	MS, KI	0.28 ± 0.01^{1*}	0.28 ± 0.01^{2*}	0.28 ± 0.01^{2*}	0.28 ± 0.01^{2*}	0.28 ± 0.01^{2*}	n.d.	0.10 ± 0.01^{1}	0.12 ± 0.02^{1*}	0.11 ± 0.01^{1*}	n.d.	n.d.	n.d.	0.10 ± 0.02^{1}	0.07 ± 0.02^{1}	0.06 ± 0.01^{1}
Benzaldehyde	MS, KI	0.25 ± 0.03^{3*}	0.25 ± 0.03^{3*}	0.25 ± 0.03^{3*}	0.25 ± 0.03^{3*}	0.25 ± 0.03*	0.29 ± 0.06	n.d.	0.26 ± 0.09	n.d.	0.30 ± 0.05	n.d.	n.d.	n.d.	n.d.	n.d.
2-Octenal, (E)-	MS, KI	n.d.	n.d.	n.d.	n.d.	n.d.	0.11 ± 0.02^{2*}	0.11 ± 0.00^{2}	0.13 ± 0.03	0.11 ± 0.02^{2}	n.d.	0.06 ± 0.00^{b1}	0.04 ± 0.00^{a1}	n.d.	0.06 ± 0.01^{ab1}	n.d.
Nonanal	MS, KI	0.35 ± 0.05^{2}	0.35 ± 0.05	0.35 ± 0.05	0.35 ± 0.05	0.35 ± 0.05	0.08 ± 0.00^{1}	n.d.	n.d.	n.d.	n.d.	n.d.	n.d.	n.d.	n.d.	n.d.
2-Decenal, (E)-	MS, KI	n.d.	n.d.	n.d.	n.d.	n.d.	n.d.	n.d.	n.d.	n.d.	n.d.	n.d.	n.d.	n.d.	n.d.	n.d.
2,4-Decadienal, (E,E)-	MS, KI	n.d.	n.d.	n.d.	n.d.	n.d.	n.d.	n.d.	n.d.	n.d.	n.d.	n.d.	n.d.	n.d.	n.d.	n.d.
2-Butyl-2-octenal	MS, KI	n.d.	n.d.	n.d.	n.d.	n.d.	0.13 ± 0.05	0.15 ± 0.01^{2}	0.17 ± 0.02	0.16 ± 0.01	n.d.	0.10 ± 0.01	0.12 ± 0.01^{1}	0.08 ± 0.03	0.14 ± 0.04	0.09 ± 0.05
<u>Alcohols</u>																
1-Penten-3-ol	MS, KI	n.d.	n.d.	n.d.	n.d.	n.d.	n.d.	n.d.	n.d.	n.d.	n.d.	0.13 ± 0.04	n.d.	n.d.	n.d.	n.d.
3-Methyl-1-butanol	MS	n.d.	n.d.	n.d.	n.d.	n.d.	n.d.	n.d.	0.07 ± 0.01	n.d.	n.d.	0.40 ± 0.21	n.d.	1.08 ± 1.12	n.d.	n.d.
1-Pentanol	MS, KI	0.41 ± 0.00	0.41 ± 0.00^{1}	0.41 ± 0.00^{1}	0.41 ± 0.00^{1}	0.41 ± 0.00^{1}	0.62 ± 0.01^{b*}	0.69 ± 0.00^{c2*}	0.63 ± 0.01^{b3*}	0.69 ± 0.01^{c3*}	0.43 ± 0.02^{a1*}	0.43 ± 0.19	0.56 ± 0.05^{12}	0.57 ± 0.02^{2}	0.58 ± 0.02^{2}	0.68 ± 0.03^{2}
1-Hexanol	MS, KI	0.53 ± 0.02^{1*}	0.53 ± 0.02^{1*}	0.53 ± 0.02*	0.53 ± 0.02^{1*}	0.53 ± 0.02^{1*}	0.62 ± 0.01^{a2*}	4.49 ± 0.11^{b2*}	0.82 ± 0.37^{a}	4.94 ± 0.13^{b3*}	0.97 ± 0.08^{a2*}	1.11 ± 0.24^{a12}	3.32 ± 0.78^{ab12}	2.07 ± 1.25^{ab}	3.56 ± 0.30^{b2}	1.88 ± 0.57^{ab12}
2-Heptanol	MS, KI	n.d.	n.d.	n.d.	n.d.	n.d.	n.d.	n.d.	n.d.	n.d.	n.d.	0.13 ± 0.03^{a}	0.47 ± 0.15^{b}	0.30 ± 0.03^{ab}	0.23 ± 0.07^{ab}	0.13 ± 0.06^{ab}

2-Hepten-1-ol, (E)-	MS	n.d.	n.d.	n.d.	n.d.	n.d.	n.d.	n.d.	n.d.	n.d.	n.d.	n.d.	n.d.	n.d.	n.d.	n.d.
1-Octen-3-ol	MS, KI	1.25 ± 0.06*	1.25 ± 0.06*	1.25 ± 0.06*	1.25 ± 0.06^{1*}	1.25 ± 0.06^{2*}	1.32 ± 0.04^{bc*}	1.46 ± 0.02^{c*}	1.37 ± 0.00^{ab*}	1.47 ± 0.11^{abc2*}	1.11 ± 0.06^{a12*}	1.44 ± 0.52	1.21 ± 0.21	1.16 ± 0.05	1.14 ± 0.05^{1}	0.88 ± 0.14^{1}
2-Ethyl-1-hexanol	MS	n.d.	n.d.	n.d.	n.d.	n.d.	n.d.	n.d.	n.d.	n.d.	n.d.	n.d.	n.d.	n.d.	n.d.	n.d.
1-Octanol	MS, KI	n.d.	n.d.	n.d.	n.d.	n.d.	0.16 ± 0.05	0.31 ± 0.03	0.20 ± 0.09	0.24 ± 0.11	0.20 ± 0.00	n.d.	n.d.	0.14 ± 0.06	n.d.	n.d.
<u>Hydrocarbons</u>																
n-Hexane	MS, KI	n.d.	n.d.	n.d.	n.d.	n.d.	0.08 ± 0.00^{2}	n.d.	0.07 ± 0.01	0.08 ± 0.02	n.d.	0.06 ± 0.01^{1}	0.16 ± 0.09	0.05 ± 0.02	0.06 ± 0.02	0.05 ± 0.00
1-Nitropropane	MS, KI	n.d.	n.d.	n.d.	n.d.	n.d.	0.10 ± 0.01	0.11 ± 0.01	0.10 ± 0.00	0.11 ± 0.00^{2}	n.d.	0.09 ± 0.01	0.09 ± 0.01	0.09 ± 0.01	0.10 ± 0.00	0.08 ± 0.01
Toluene	MS, KI	n.d.	n.d.	n.d.	n.d.	n.d.	n.d.	0.07 ± 0.01^{a*}	0.08 ± 0.01^{ab}	0.11 ± 0.02^{b*}	n.d.	0.05 ± 0.01	0.06 ± 0.01	0.06 ± 0.01	n.d.	0.05 ± 0.00
Octane	MS, KI	n.d.	n.d.	n.d.	n.d.	n.d.	n.d.	n.d.	n.d.	n.d.	n.d.	n.d.	n.d.	n.d.	0.05 ± 0.01	n.d.
2-Octene	MS, KI	n.d.	n.d.	n.d.	n.d.	n.d.	n.d.	n.d.	n.d.	n.d.	n.d.	n.d.	n.d.	n.d.	n.d.	n.d.
1-Nitrobutane	MS, KI	n.d.	n.d.	n.d.	n.d.	n.d.	0.16 ± 0.00^{2}	0.16 ± 0.01	0.15 ± 0.01	0.18 ± 0.01^{2}	n.d.	0.15 ± 0.01^{1}	0.16 ± 0.02	0.16 ± 0.02	0.14 ± 0.01^{1}	0.13 ± 0.01
1-Nitropentane	MS, KI	4.16 ± 0.24^{2}	4.16 ± 0.24^{2}	4.16 ± 0.24^{2}	4.16 ± 0.24^{2}	4.16 ± 0.24^{2}	3.81 ± 0.19^{b2*}	3.79 ± 0.04^{b2*}	3.61 ± 0.13^{b12*}	3.85 ± 0.20^{b2*}	3.06 ± 0.15^{a1*}	2.93 ± 0.14^{1}	3.02 ± 0.19^{1}	3.03 ± 0.36^{1}	2.86 ± 0.08^{1}	2.81 ± 0.13^{1}
2-Pentylfuran	MS, KI	1.92 ± 0.34	1.92 ± 0.34^{2}	1.92 ± 0.34^{2}	1.92 ± 0.34	1.92 ± 0.34	0.60 ± 0.15^{ab}	0.41 ± 0.03^{a1}	0.85 ± 0.14^{bc1*}	0.84 ± 0.03^{bc*}	0.96 ± 0.05^{c*}	0.68 ± 0.14	0.86 ± 0.00^{12}	0.66 ± 0.12^{1}	0.93 ± 0.17	0.63 ± 0.41
2,2,4,6,6-Pentamethylheptane	MS	n.d.	n.d.	n.d.	n.d.	n.d.	n.d.	n.d.	n.d.	n.d.	n.d.	n.d.	n.d.	n.d.	n.d.	n.d.
1-Nitrohexane	MS, KI	2.71 ± 0.23^{3}	2.71 ± 0.23^{2}	2.71 ± 0.23^{3}	2.71 ± 0.23^{3}	2.71 ± 0.23^{3}	1.96 ± 0.17^{2}	1.79 ± 0.08^{12}	1.87 ± 0.11^{2}	1.85 ± 0.15^{2}	1.64 ± 0.15^{2}	1.23 ± 0.08^{1}	1.30 ± 0.04^{1}	1.43 ± 0.03^{1}	1.22 ± 0.04^{1}	1.22 ± 0.04^{1}

<u>Ketones</u>																
2,3-Butanedione	MS	n.d.	n.d.	n.d.	n.d.	n.d.	n.d.	n.d.	n.d.	n.d.	n.d.	n.d.	n.d.	n.d.	n.d.	n.d.
3-Hydroxybutanone	MS, KI	n.d.	n.d.	n.d.	n.d.	n.d.	n.d.	n.d.	n.d.	n.d.	n.d.	n.d.	n.d.	n.d.	n.d.	n.d.
2-Methyl-3-pentanone	MS	n.d.	n.d.	n.d.	n.d.	n.d.	n.d.	n.d.	n.d.	n.d.	n.d.	n.d.	n.d.	n.d.	n.d.	n.d.
2-Methyl-1-penten-3-one	MS	n.d.	n.d.	n.d.	n.d.	n.d.	n.d.	n.d.	n.d.	n.d.	n.d.	n.d.	n.d.	n.d.	n.d.	n.d.
2-Hexanone	MS, KI	n.d.	n.d.	n.d.	n.d.	n.d.	0.24 ± 0.01*	0.24 ± 0.01^{2*}	0.24 ± 0.01*	0.24 ± 0.00^{2*}	n.d.	0.24 ± 0.05	0.18 ± 0.01^{1}	0.18 ± 0.02	0.19 ± 0.01^{1}	0.20 ± 0.02
2-Heptanone	MS, KI	5.45 ± 0.30*	5.45 ± 0.30^{2*}	5.45 ± 0.30^{2*}	5.45 ± 0.30*	5.45 ± 0.30^{2*}	5.22 ± 0.22^{b*}	5.07 ± 0.06^{b2*}	4.96 ± 0.32^{b2*}	5.14 ± 0.22^{b*}	4.32 ± 0.22^{a1*}	4.39 ± 0.88	3.12 ± 0.13^{1}	3.82 ± 0.30^{1}	3.52 ± 0.10	3.93 ± 0.27^{1}
3-Ethylcyclopentanone	MS	n.d.	n.d.	n.d.	n.d.	n.d.	0.09 ± 0.01	0.09 ± 0.01	0.08 ± 0.01	0.09 ± 0.00	n.d.	0.10 ± 0.02	0.07 ± 0.02	0.08 ± 0.02	0.08 ± 0.01	0.07 ± 0.02
1-Hepten-3-one	MS	n.d.	n.d.	n.d.	n.d.	n.d.	n.d.	n.d.	n.d.	n.d.	n.d.	n.d.	n.d.	n.d.	n.d.	n.d.
3-Octanone	MS, KI	n.d.	n.d.	n.d.	n.d.	n.d.	n.d.	n.d.	n.d.	n.d.	n.d.	0.41 ± 0.18	0.58 ± 0.04	n.d.	0.53 ± 0.01	0.55 ± 0.20
2-Octanone	MS	0.68 ± 0.06^{1*}	0.68 ± 0.06^{1*}	0.68 ± 0.06^{1*}	0.68 ± 0.06^{1*}	0.68 ± 0.06^{2*}	0.64 ± 0.01^{ab1*}	0.95 ± 0.03^{c2*}	0.68 ± 0.09^{b1*}	0.95 ± 0.12^{c2*}	0.45 ± 0.02^{a1*}	0.90 ± 0.08^{2}	0.75 ± 0.07^{1}	0.93 ± 0.11^{2}	0.85 ± 0.04^{12}	0.75 ± 0.28^{12}
3-Octen-2-one	MS, KI	n.d.	n.d.	n.d.	n.d.	n.d.	n.d.	n.d.	n.d.	n.d.	n.d.	n.d.	n.d.	n.d.	n.d.	n.d.
3,5-Octadien-2-one	MS, KI	n.d.	n.d.	n.d.	n.d.	n.d.	0.14 ± 0.01^{b2}	0.13 ± 0.01^{b2}	0.13 ± 0.01^{ab2}	0.11 ± 0.01^{a2}	n.d.	0.06 ± 0.00^{1}	0.05 ± 0.01^{1}	0.07 ± 0.01^{1}	0.06 ± 0.00^{1}	0.06 ± 0.01
2-Nonanone	MS, KI	0.81 ± 0.12*	0.81 ± 0.12^{2*}	0.81 ± 0.12^{2*}	0.81 ± 0.12^{2*}	0.81 ± 0.12^{2*}	0.53 ± 0.04*	0.55 ± 0.00^{12*}	0.52 ± 0.03^{12*}	0.60 ± 0.06^{12*}	0.43 ± 0.04^{12*}	0.47 ± 0.35	0.27 ± 0.01^{1}	0.30 ± 0.04^{1}	0.30 ± 0.03^{1}	0.29 ± 0.03^{1}
2-Decanone	MS, KI	0.26 ±	0.26 ±	0.26 ±	0.26 ±	0.26 ± 0.04^{2}	0.15 ± 0.02^{1}	0.14 ± 0.01^{1}	0.16 ± 0.00^{1}	0.17 ± 0.02^{1*}	n.d.	0.13 ± 0.01^{1}	0.12 ± 0.02^{1}	0.10 ± 0.02^{1}	0.11 ± 0.00^{1}	0.08 ± 0.04^{1}

		0.04[2]	0.04[2]	0.04[2]	0.04[2]											
<u>Other compounds</u>																
Phenol, 2,4-bis(1,1-dimethylethyl)-	MS	n.d.	n.d.	n.d.	n.d.	n.d.	0.08 ± 0.02	0.07 ± 0.01	0.09 ± 0.02	0.33 ± 0.28	n.d.	0.05 ± 0.01	0.07 ± 0.00	0.07 ± 0.03	0.07 ± 0.03	0.12 ± 0.04

b)

Name	id.	D0 CG13	D0 CG 15	D0 CG 28	D0 CG 40	D0 CG0	D1 CG13	D1 CG15	D1 CG28	D1 CG40	D1 CG0
Acids											
Acetic acid	MS	n.d.	n.d.	n.d.	n.d.	n.d.	0.41 ± 0.29^{ab}	0.05 ± 0.00^{a}	0.25 ± 0.11^{ab}	n.d.	0.63 ± 0.19^{b}
3-Methyl-butanoic acid	MS	n.d.	n.d.	n.d.	n.d.	n.d.	n.d.	n.d.	0.13 ± 0.03	n.d.	n.d.
2-Methyl-butanoic acid	MS	n.d.	n.d.	n.d.	n.d.	n.d.	n.d.	n.d.	0.05 ± 0.01	n.d.	0.14 ± 0.15
Aldehydes											
3-Methylbutanal	MS, KI	n.d.	n.d.	n.d.	n.d.	n.d.	1.24 ± 0.96	0.11 ± 0.09	0.60 ± 0.29*	n.d.	0.06 ± 0.00
2-Methylbutanal	MS, KI	n.d.	n.d.	n.d.	n.d.	n.d.	0.07 ± 0.03	n.d.	0.06 ± 0.03*	n.d.	n.d.
Pentanal	MS, KI	0.68 ± 0.01*	0.68 ± 0.01*	0.68 ± 0.01*	0.68 ± 0.01*	0.68 ± 0.01*	n.d.	n.d.	n.d.	n.d.	n.d.
Hexanal	MS, KI	13.85 ± 0.31^{2}*	13.85 ± 0.31*	13.85 ± 0.31^{2}*	13.85 ± 0.31^{2}*	13.85 ± 0.31^{2}*	0.38 ± 0.13^{c1}*	n.d.	0.13 ± 0.01^{ab1}	0.05 ± 0.01^{a1}*	0.29 ± 0.06^{bc1}*
2-Ethyl-2-butenal	MS	n.d.	n.d.	n.d.	n.d.	n.d.	n.d.	n.d.	n.d.	n.d.	n.d.
2-Hexenal, (E)-	MS, KI	n.d.	n.d.	n.d.	n.d.	n.d.	n.d.	n.d.	n.d.	n.d.	n.d.
Heptanal	MS, KI	0.92 ± 0.09	0.92 ± 0.09	0.92 ± 0.09	0.92 ± 0.09	0.92 ± 0.09	n.d.	n.d.	n.d.	n.d.	n.d.
2-Heptenal, (Z)-	MS, KI	1.33 ± 0.31*	1.33 ± 0.31*	1.33 ± 0.31^{2}*	1.33 ± 0.31^{2}*	1.33 ± 0.31*	2.02 ± 0.42^{b}	0.79 ± 0.46^{a}	0.68 ± 0.09^{a1}*	0.64 ± 0.14^{a1}*	1.16 ± 0.33^{ab}
Benzaldehyde	MS, KI	0.40 ± 0.05*	0.40 ± 0.05*	0.40 ± 0.05*	0.40 ± 0.05*	0.40 ± 0.05*	n.d.	n.d.	n.d.	n.d.	n.d.

2-Octenal, (E)-	MS, KI	0.35 ± 0.10	0.35 ± 0.10^{2}	0.35 ± 0.10^{2}	0.35 ± 0.10^{2}	0.35 ± 0.10^{2}	0.15 ± 0.01*	0.09 ± 0.04^{1}	0.08 ± 0.02^{1}	0.08 ± 0.02^{1}	0.14 ± 0.03^{1}
Nonanal	MS, KI	0.48 ± 0.09^{2}	0.48 ± 0.09	0.48 ± 0.09	0.48 ± 0.09	0.48 ± 0.09	0.06 ± 0.02^{1}	n.d.	n.d.	n.d.	n.d.
2-Decenal, (E)-	MS, KI	n.d.	n.d.	n.d.	n.d.	n.d.	0.08 ± 0.03^{ab}	0.07 ± 0.03^{ab}	n.d.	0.05 ± 0.00^{a}	0.13 ± 0.04^{b}
2,4-Decadienal, (E,E)-	MS, KI	n.d.	n.d.	n.d.	n.d.	n.d.	n.d.	n.d.	n.d.	n.d.	0.05 ± 0.01
2-Butyl-2-octenal	MS, KI	n.d.	n.d.	n.d.	n.d.	n.d.	n.d.	n.d.	n.d.	n.d.	n.d.
Alcohols											
1-Penten-3-ol	MS, KI	n.d.	n.d.	n.d.	n.d.	n.d.	n.d.	n.d.	n.d.	n.d.	n.d.
3-Methyl-1-butanol	MS	n.d.	n.d.	n.d.	n.d.	n.d.	0.69 ± 0.19^{b}	0.22 ± 0.16^{ab}	0.18 ± 0.24^{ab}	0.04 ± 0.01^{a}	0.09 ± 0.00^{ab}
1-Pentanol	MS, KI	n.d.	n.d.	n.d.	n.d.	n.d.	0.08 ± 0.00*	0.17 ± 0.07*	0.11 ± 0.00*	0.18 ± 0.05*	0.14 ± 0.03*
1-Hexanol	MS, KI	0.16 ± 0.02*	0.16 ± 0.02^{1}*	0.16 ± 0.02*	0.16 ± 0.02^{1}*	0.16 ± 0.02^{1}*	0.21 ± 0.08^{a}*	2.09 ± 0.65^{ab2}*	0.13 ± 0.03^{a}	1.84 ± 0.07^{b2}*	1.72 ± 0.33^{b2}*
2-Heptanol	MS, KI	n.d.	n.d.	n.d.	n.d.	n.d.	n.d.	n.d.	n.d.	n.d.	n.d.
2-Hepten-1-ol, (E)-	MS	n.d.	n.d.	n.d.	n.d.	n.d.	0.24 ± 0.14	n.d.	n.d.	n.d.	0.24 ± 0.08
1-Octen-3-ol	MS, KI	2.78 ± 0.26^{1}*	2.78 ± 0.26^{1}*	2.78 ± 0.26*	2.78 ± 0.26*	2.78 ± 0.26*	3.61 ± 0.17^{2}*	3.28 ± 0.16^{2}*	3.25 ± 0.15*	3.22 ± 0.07*	3.19 ± 0.22*
2-Ethyl-1-hexanol	MS	n.d.	n.d.	n.d.	n.d.	n.d.	n.d.	0.20 ± 0.04	0.08 ± 0.02	0.10 ± 0.03	0.07 ± 0.00
1-Octanol	MS, KI	0.19 ± 0.02	0.19 ± 0.02	0.19 ± 0.02	0.19 ± 0.02	0.19 ± 0.02	0.19 ± 0.14	0.26 ± 0.17	0.29 ± 0.14	0.23 ± 0.08	0.25 ± 0.12
Hydrocarbons											
n-Hexane	MS, KI	n.d.	n.d.	n.d.	n.d.	n.d.	0.06 ± 0.01	0.05 ± 0.01	n.d.	n.d.	n.d.

1-Nitropropane	MS, KI	n.d.	n.d.	n.d.	n.d.	n.d.	n.d.	n.d.	n.d.	n.d.	n.d.
Toluene	MS, KI	n.d.	n.d.	n.d.	n.d.	n.d.	0.10 ± 0.02	0.11 ± 0.01*	0.07 ± 0.03	0.06 ± 0.00*	0.10 ± 0.03
Octane	MS, KI	n.d.	n.d.	n.d.	n.d.	n.d.	0.13 ± 0.01^{ab}	0.27 ± 0.06^{c}	0.08 ± 0.03^{a}	0.18 ± 0.03^{b}	0.16 ± 0.02^{ab}
2-Octene	MS, KI	n.d.	n.d.	n.d.	n.d.	n.d.	0.08 ± 0.01^{a}	0.15 ± 0.02^{b}	n.d.	0.12 ± 0.02^{b}	0.06 ± 0.01^{a}
1-Nitrobutane	MS, KI	n.d.	n.d.	n.d.	n.d.	n.d.	n.d.	n.d.	n.d.	n.d.	n.d.
1-Nitropentane	MS, KI	n.d.	n.d.	n.d.	n.d.	n.d.	0.12 ± 0.01*	0.06 ± 0.01*	0.05 ± 0.02*	0.06 ± 0.01*	0.07 ± 0.03*
2-Pentylfuran	MS, KI	2.49 ± 0.53^{2}	2.49 ± 0.53^{2}	2.49 ± 0.53^{2}	2.49 ± 0.53^{2}	2.49 ± 0.53^{2}	0.46 ± 0.15^{b1}	0.44 ± 0.11^{ab1}	0.44 ± 0.07^{ab1}*	0.47 ± 0.13^{b1}*	0.14 ± 0.08^{a1}*
2,2,4,6,6-Pentamethylheptane	MS	0.54 ± 0.16^{2}	0.54 ± 0.16^{2}	0.54 ± 0.16^{2}	0.54 ± 0.16^{2}	0.54 ± 0.16	0.10 ± 0.01^{1}	0.16 ± 0.01^{1}	0.24 ± 0.10^{1}	0.19 ± 0.09^{1}	0.29 ± 0.40
1-Nitrohexane	MS, KI	n.d.	n.d.	n.d.	n.d.	n.d.	n.d.	n.d.	n.d.	n.d.	n.d.
Ketones											
2,3-Butanedione	MS	n.d.	n.d.	n.d.	n.d.	n.d.	0.06 ± 0.02	n.d.	0.09 ± 0.04	n.d.	0.13 ± 0.04
3-Hydroxybutanone	MS, KI	n.d.	n.d.	n.d.	n.d.	n.d.	0.61 ± 0.16	n.d.	0.47 ± 0.06	0.06 ± 0.02	1.05 ± 0.69
2-Methyl-3-pentanone	MS	n.d.	n.d.	n.d.	n.d.	n.d.	0.06 ± 0.00	0.10 ± 0.04	0.07 ± 0.02	0.06 ± 0.01	0.07 ± 0.01
2-Methyl-1-penten-3-one	MS	n.d.	n.d.	n.d.	n.d.	n.d.	0.10 ± 0.02^{b}	n.d.	0.04 ± 0.00^{a}	n.d.	0.11 ± 0.01^{b}
2-Hexanone	MS, KI	n.d.	n.d.	n.d.	n.d.	n.d.	0.05 ± 0.00^{ab}*	0.05 ± 0.00^{b}*	0.05 ± 0.00^{a}*	0.05 ± 0.00^{ab}*	0.05 ± 0.00^{ab}
2-Heptanone	MS, KI	1.75 ± 0.01^{2}*	1.75 ± 0.01^{2}*	1.75 ± 0.01^{2}*	1.75 ± 0.01^{2}*	1.75 ± 0.01*	1.26 ± 0.28^{1}*	1.31 ± 0.12^{1}*	1.20 ± 0.18^{1}*	1.27 ± 0.02^{1}*	1.38 ± 0.05*
3-Ethylcyclopentanone	MS	n.d.	n.d.	n.d.	n.d.	n.d.	n.d.	n.d.	n.d.	n.d.	n.d.
1-Hepten-3-one	MS	n.d.	n.d.	n.d.	n.d.	n.d.	0.08 ± 0.01	n.d.	n.d.	n.d.	0.04 ± 0.01

3-Octanone	MS, KI	n.d.	n.d.	n.d.	n.d.	n.d.	0.17 ± 0.06^{ab}	0.70 ± 0.45^{b}	n.d.	0.09 ± 0.01^{a}	0.15 ± 0.07^{ab}
2-Octanone	MS	0.19 ± 0.05*	0.19 ± 0.05^{1}*	0.19 ± 0.05*	0.19 ± 0.05*	0.19 ± 0.05*	0.25 ± 0.04^{ab}*	0.42 ± 0.03^{b2}*	0.18 ± 0.08^{a}*	0.25 ± 0.04^{ab}*	0.20 ± 0.12^{a}*
3-Octen-2-one	MS, KI	0.25 ± 0.05^{2}	0.25 ± 0.05	0.25 ± 0.05	0.25 ± 0.05	0.25 ± 0.05^{2}	0.14 ± 0.02^{1}	n.d.	n.d.	n.d.	0.11 ± 0.01^{1}
3,5-Octadien-2-one	MS, KI	n.d.	n.d.	n.d.	n.d.	n.d.	n.d.	n.d.	n.d.	n.d.	n.d.
2-Nonanone	MS, KI	0.18 ± 0.01^{2}*	0.18 ± 0.01*	0.18 ± 0.01^{2}*	0.18 ± 0.01^{2}*	0.18 ± 0.01^{2}*	0.10 ± 0.02^{1}*	0.12 ± 0.04*	0.09 ± 0.01^{1}*	0.12 ± 0.01^{1}*	0.08 ± 0.02^{1}*
2-Decanone	MS, KI	n.d.	n.d.	n.d.	n.d.	n.d.	n.d.	n.d.	n.d.	0.04 ± 0.00*	n.d.
Other compounds											
Phenol, 2,4-bis(1,1-dimethylethyl)-	MS	n.d.	n.d.	n.d.	n.d.	n.d.	0.07 ± 0.02	0.12 ± 0.05	0.09 ± 0.02	0.07 ± 0.05	0.07 ± 0.03

Data is displayed as mean ± standard deviation (x10^6). Different superscript letters (a-c) and numbers (1-3) indicate significant differences among strains within the same day of fermentation, and among days within the same strain, respectively, and asterisks (*) denote differences between protein matrices for a given strain and sampling time ($P < 0.05$).
